# A blastoporal organizer in a ctenophore

**DOI:** 10.1038/s41586-026-10643-z

**Published:** 2026-06-17

**Authors:** Stanislav Kremnyov, Tatiana Lebedeva, Grigory Genikhovich, Andreas Hejnol

**Affiliations:** 1 Institute of Zoology and Evolutionary Research, Faculty of Biological Sciences, Friedrich Schiller University Jena, Jena, Germany; 2 Department of Neurosciences and Developmental Biology, Faculty of Life Sciences, University of Vienna, Vienna, Austria

**Keywords:** Evolutionary developmental biology, Embryology

## Abstract

In an iconic experiment in 1924, Hilde Mangold and Hans Spemann established that the dorsal blastopore lip of amphibian embryos functions as an organizer and induces a secondary body axis when transplanted into a host embryo^1^. This discovery demonstrated that specific embryonic regions can regulate embryonic patterning and lead to the establishment of an entire body axis. Subsequent studies have revealed that cnidarians, the sister group to Bilateria, also possess a blastoporal embryonic organizer^2,3^. However, the evolutionary origin of the organizer remains unclear. Here we report that the blastopore lip of the ctenophore *Mnemiopsis leidyi*, a member of the evolutionary sister group to all other metazoans^4,5^, exhibits organizer activity. We show that transplanted fragments of blastopore lip tissue from *M. leidyi* gastrula induce secondary pharynx and mouth formation. Moreover, transphyletic transplantation experiments show that the blastopore lip of *M. leidyi* leads to the generation of a secondary body axis in embryos of the cnidarian *Nematostella vectensis*. Organizer function in *M. leidyi* requires both β-catenin and TGFβ signalling, and the TGFβ-family ligands probably provide this inductive capacity. These findings reveal the deep homology of the blastoporal organizer in ctenophores, cnidarians and vertebrates, implying the ancestral organizer role of the blastopore lip. We propose that the emergence of the organizer was an essential innovation that facilitated the change from the temporal cell differentiation of unicellular relatives to the spatial cell differentiation of the first multicellular embryo.

## Abstract (web summary)

Experiments using the comb jelly *Mnemiopsis leidyi* and the sea anemone *Nematostella vectensis* reveal that the emergence of a core signalling pathway may have been a key innovation enabling the transition to multicellularity in animals.

## Main

**Fig. 1:**
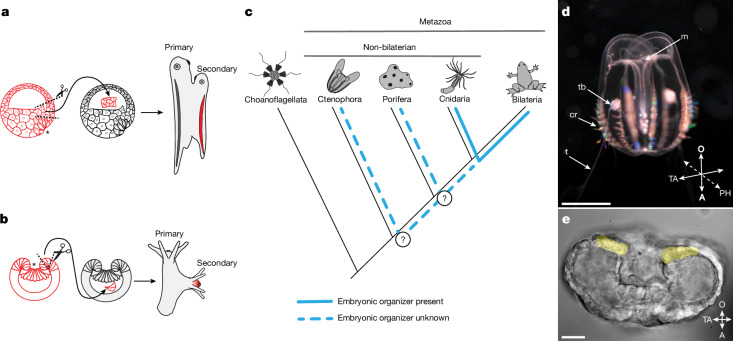
Embryonic organizers in different metazoan groups. **a**,**b**, Schematics of embryonic organizer transplantation in amphibians (**a**) and cnidarians (anthozoans) (**b**). In both cases, transplantation of the blastopore lip fragment from one embryo to another induces a secondary axis involving host tissue. **c**, Presence of an embryonic organizer in different metazoan groups. The embryonic organizer has so far been described and studied only in bilaterians and their sister group cnidarians. However, it remains unknown whether an embryonic organizer exists in Porifera or Ctenophora; therefore, it is unclear when the embryonic organizer emerged in animal evolution. **d**, Cydippid stage of *M. leidyi* (Ctenophora). Scale bar, 2.5 mm. **e**, *M. leidyi* embryo at the gastrula stage. Cells surrounding the blastopore are highlighted yellow. Scale bar, 20 μm. Images in **d** and **e** are representative images from normal wild-type *M. leidyi* at the indicated developmental stages. Such normal morphology was consistently observed in all examined specimens. Asterisks (**a**,**b**,**e**) indicate the blastopore. A, aboral; cr, comb row; m, mouth; O, oral; PH, pharyngeal axis; t, tentacle; TA, tentacular axis; tb, tentacle bud.

The concepts of the embryonic organizer and induction were introduced and developed by Hilde Mangold and Hans Spemann based on their landmark experiments in the 1920s^1,6^. Their studies on transplantation of the dorsal blastopore lip of amphibian embryos demonstrated that this tissue has the capacity to induce the formation of a secondary body axis by recruiting neighbouring host cells (Fig. 1a). The secondary axis is formed through interactions between the transplanted cells and the host embryo tissues, and the data highlighted the critical role of the organizer in directing host tissue differentiation and morphogenesis. This experiment provided clear evidence that specific cell populations in embryos can influence the differentiation of neighbouring cells. Subsequent comparative evolutionary studies revealed the presence of an embryonic organizer in a non-bilaterian animal, the cnidarian *N. vectensis*^2,3^. Transplanting a section of the blastopore lip from an early gastrula of *N. vectensis* to another embryo at the same stage of development results in the induction of a secondary oral–aboral axis (Fig. 1b). The central role of WNT–β-catenin signalling in driving the organizing activity of cnidarian and vertebrate blastopore lips suggest the potential homology of these structures. Despite extensive research on the embryonic organizer in bilaterians, particularly chordates (the zebrafish *Danio rerio* and the frog *Xenopus laevis*)^7,8^, and their sister group cnidarians (*N. vectensis*)^2,3^, the evolutionary history of the embryonic organizer remains unsolved. It is still unclear whether embryonic organizers exist in other non-bilaterian animals predating the cnidaria–bilaterian separation, like sponges (Porifera) and comb jellies (Ctenophora) (Fig. 1c). Ctenophores are of particular interest because they are considered to occupy a key phylogenetic position as a sister group to all other multicellular animals^4,5^. A ctenophore embryonic organizer, if homologous to those in cnidarians and bilaterians, would suggest that an organizer emerged alongside the advent of multicellularity.

## Ctenophore blastopore lip is an organizer

**Fig. 2:**
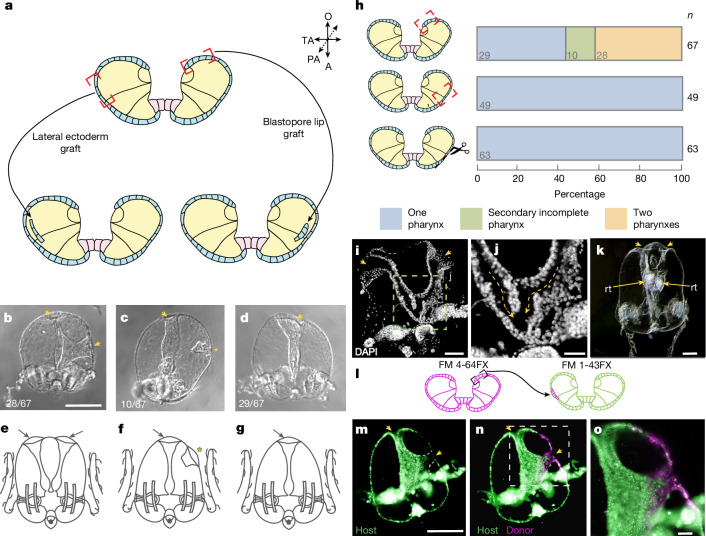
Organizer activity of the blastopore lip of a *M. leidyi* gastrula. **a**, Schematic of the grafting experiment: transplantation of the blastoporal lip tissue or transplantation of lateral ectoderm. **b**–**g**, Images (top row) and schematics (bottom row) of the phenotypes obtained in transplantation experiment: two pharynxes (**b**,**e**), a secondary incomplete pharynx (**c**,**f**) or one pharynx (**d**,**g**). Arrowheads indicate a mouth opening with a fully formed pharynx in the cydippid. The asterisk indicates the formation of a mouth-like structure without a pharynx. Numbers in the bottom left present the fraction of embryos displaying this phenotype in the blastoporal lip tissue transplantation experiment. **h**, Transplantation of only the blastopore lip of a *M. leidyi* embryo can induce ectopic development of the fully formed axial structures or an ectopic secondary incomplete axis (top). By contrast, transplantation of the lateral ectoderm (middle) or incision without transplantation (bottom) results in the formation of a cydippid with only one body axis. Stacked bars show pooled proportions. **i**,**j**, Images of two fully formed pharynxes fused distally towards the infundibulum (*n* = 3). **i**, Arrowheads indicate the mouth openings. **j**, Arrows indicate the direction of fusion of the two pharynxes. **k**, Two fully developed pharynxes are visible, each with an ingested rotifer (rt). Arrowheads denote the mouth openings, and arrows indicate rotifers located in the pharyngeal cavities (*n* = 4). **l**, Schematic of the experiment for transplanting *M. leidyi* blastopore lip labelled with the vital dye FM4-64FX into a host embryo labelled with FM1-43FX. **m**–**o**, Images showing host tissues (**m**), and how the grafted blastopore contributed to the formation of an ectopic pharynx with integration of host tissues (**n**); close-up of the ectopic pharynx and mouth (**o**) (*n* = 5). Arrowheads denote the mouth openings. Scale bars, 250 μm (**b**–**d**,**m**,**n**), 100 μm (**k**), 50 μm (**i**,**o**), 25 μm (**j**).

**Extended Data Fig. 1:**
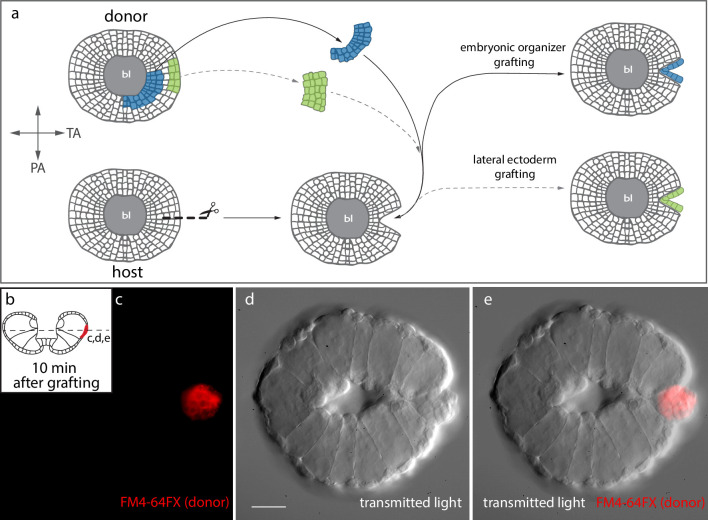
Grafting of donor embryonic tissues into the lateral side of *Mnemiopsis leidyi* embryos. **a**, Schematic overview of the grafting experiment. All embryos are oriented in oral view up. The approximate size of the blastoporal region used for grafting is indicated in blue. The region of lateral ectoderm used for grafts is marked in green. The dashed line in host embryo indicates the cut made in the host embryo, which creates a V-shaped wound serving as a pocket for the graft. Solid arrows indicate transplantation of the blastoporal organizer region, dashed arrows indicate transplantation of lateral ectoderm. **b–e**, Host embryo 10 min after transplantation of donor blastoporal tissue. Donor tissue is labelled with the vital lipophilic dye FM4-64FX. In **b**, the scheme shows the level of the focal plane for the imaged embryo with graft. This experiment was independently repeated n = 4 times with similar results. bl, blastopore; PA, pharyngeal axis; TA, tentacular axis. Scale bar, 20 µm.

To analyse the inductive capacity of different parts of the *M. leidyi* gastrula embryo, we transplanted blastopore lip tissue and lateral ectoderm to host embryos of the same stage of development (Fig. 2a and Extended Data Fig. 1). In our experiments, 41.8% (28 out of 67) of blastopore lip transplantations resulted in the formation of a complete secondary pharynx with a mouth opening (Fig. 2b). Moreover, 14.9% (10 out of 67) of embryos formed a mouth-like structure without a developed pharynx (Fig. 2c), and 43.3% (29 out of 67) developed normally (Fig. 2d). We did not observe duplication of any other anatomical structures. By contrast, embryos that received lateral ectoderm grafts (*n* = 49) never formed secondary pharynxes or mouth openings. Similarly, after control incisions without grafting (*n *= 63), we did not observe the formation of any additional structures (Fig. 2e–h). In cases when the ectopic pharynx was fully developed, the primary and secondary induced pharynxes fused together before connecting to the ‘stomach’ (infundibulum) and merging into a single digestive system (Fig. 2i,j). Both the primary and the ectopically induced mouths were fully functional and capable of capturing food, such as rotifers, and delivering it into the shared gastrovascular system (Fig. 2k).

## Organizer transplants recruit host tissue

**Extended Data Fig. 2:**
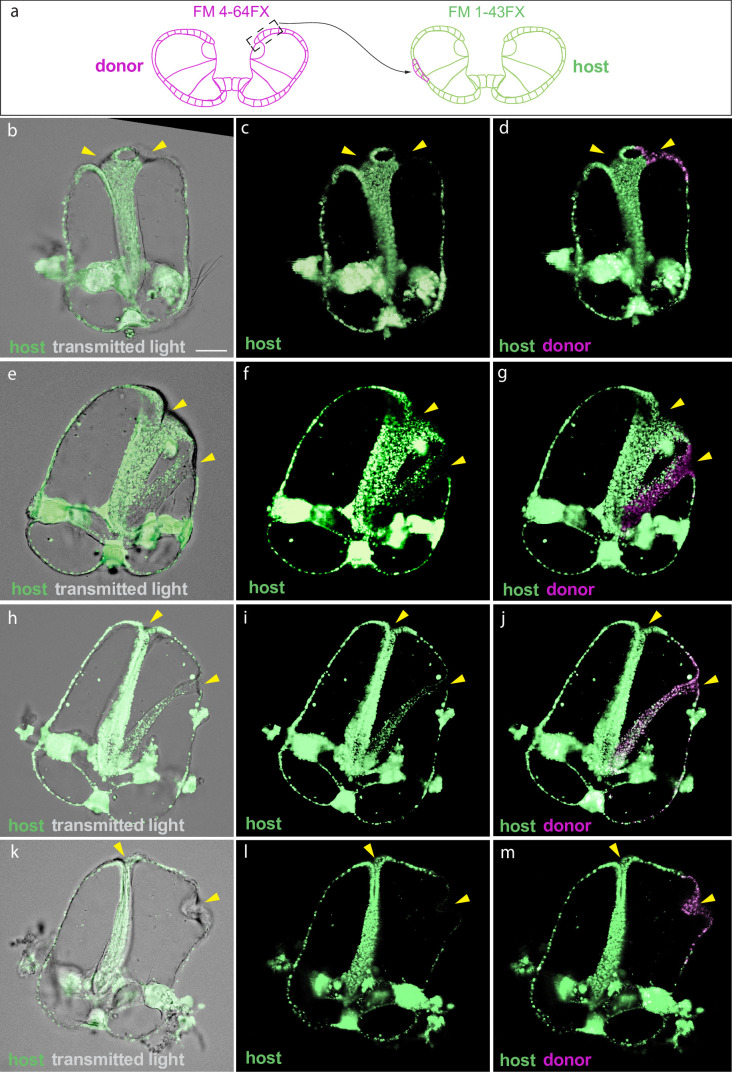
Variability in the distribution of grafted blastopore tissue contributing to ectopic pharynx formation with integration of host cells. **a**, Scheme of the experiment on transplanting the *M. leidyi* blastopore lip labeled with the vital dye FM4-64FX into a host embryo labeled with FM1-43FX. **b**–**d**, Ectopic pharynx composed of donor and host tissues with a sharp boundary between them. **e**–**g**, Ectopic pharynx in which one pharyngeal wall contains host-derived cells. **h–j**, Induced pharynx composed of a uniformly mixed donor–host cell population. **k**–**m**, Mouth-like structure without a developed pharynx consisting entirely of grafted tissue showing case of failed induction. This transplantation experiment was performed n = 34 times in total, and each phenotype shown was observed at least three times. Yellow arrowheads denote the mouth openings. The scale bar in panel **b** (100 µm) also applies to panels **c–m**.

An essential characteristic of an organizer is to recruit surrounding host tissues to form induced structures after transplantation^1,9^. To explore this effect in our experimental model, we transplanted pieces of the blastopore lip, which were labelled with the vital membrane dye FM4-64FX, into FM1-43FX-labelled host gastrula embryos (Fig. 2l and Extended Data Fig. 2a). In transplantations that resulted in the induction of a fully developed ectopic pharynx, the pharyngeal tissue was composed of a mix of both host cells (FM1-43FX-labelled) and transplanted cells (FM4-64FX-labelled) (Fig. 2m–o).

However, the distribution of donor and host cells in the induced pharynx varied. In one instance, the ectopic pharynx consisted of donor and host tissues with a sharp boundary between them (Extended Data Fig. 2b–d). Alternatively, only one wall of the ectopic pharynx contained host-derived cells (Extended Data Fig. 2e–g) or the induced pharynx was composed of a uniformly mixed population of donor and host cells (Extended Data Fig. 2h–j). Notably, host cells were never observed in the area of the ectopic mouth opening (Fig. 2m–o and Extended Data Fig. 2b–j). This kind of split between donor and host contributions is also seen in classical Mangold–Spemann grafts. That is, donor cells largely contribute to the axial midline (for example, the ectopic notochord is almost entirely donor-derived), whereas surrounding parts of the induced axis often contain a mixture of donor and host cells^1^. Here we determined that the phenotype described as a ‘mouth-like structure without a developed pharynx’ (Fig. 2c,f) results from failed induction, as this structure is composed exclusively of donor tissue (Extended Data Fig. 2k–m).

**Extended Data Fig. 3:**
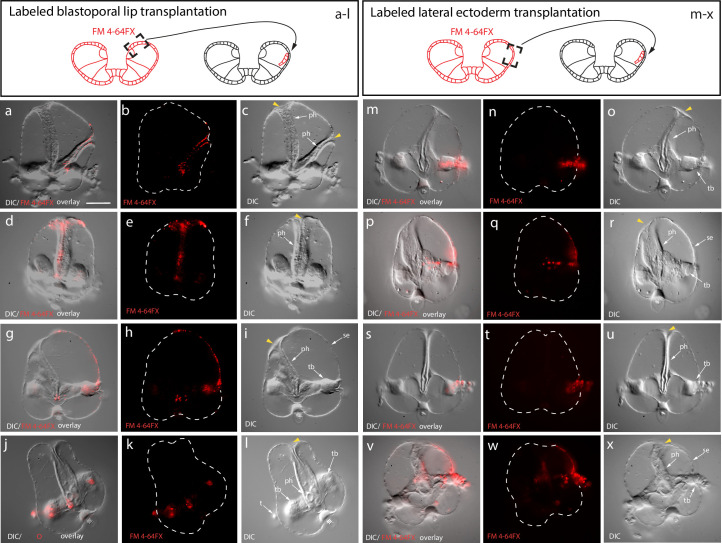
Variability in the distribution of grafted tissue labeled with the vital dye FM4-64FX in the host animal. **a**–**l**, Transplantation of labeled gastrula blastopore lip. **m**–**x**, Transplantation of labeled gastrula lateral ectoderm. **a**–**c**, The grafted blastopore tissue contributed to the formation of an ectopic pharynx with the integration of host tissues. **d**–**f**, Incorporation of grafted blastopore cells into host pharyngeal tissue. **g**–**i**, Integration of grafted blastopore cells into host tissues of superficial epithelium, tentacle bulb and pharynx. **j**–**l**, Integration of blastopore cells into host tissues of tentacle, tentacle bulb and pharynx. **m**–**x**, Integration of grafted lateral ectoderm into the host tissues of tentacle, tentacle bulb pharynx and superficial epithelium. The transplantation experiment shown in panels **a**–**l** was performed n = 41 times, and that shown in panels **m**–**x** was performed n = 29 times each phenotype shown was observed at least three times. All cydippids are at 2 days post-fertilization. Yellow arrowheads indicate mouth opening with fully formed pharynx in the cydippid. ph – pharynx, se – superficial epithelium, t – tentacle. tb – tentacle bulb. The scale bar in panel **a** (100 µm) also applies to panels **b**–**x**.

For transplantations in which the blastopore lip graft did not induce an ectopic pharynx, we selectively labelled donor cells with FM4-64FX to trace the distribution of the transplanted tissues (Extended Data Fig. 3). In these embryos, we observed considerable variability in the distribution of donor-derived tissue. Donor cells were incorporated entirely in the primary pharynx (Extended Data Fig. 3d–f), scattered across the superficial epithelium (Extended Data Fig. 3g–i) or integrated into the tentacle bulb and tentacles (Extended Data Fig. 3g–l).

Thus, the formation of the secondary pharynx and mouth following blastopore lip transplantation in ctenophores involves the recruitment of host cells into the ectopically developing structure, which is a hallmark of a bona fide organizer.

## Transphyletic axis induction

**Fig. 3:**
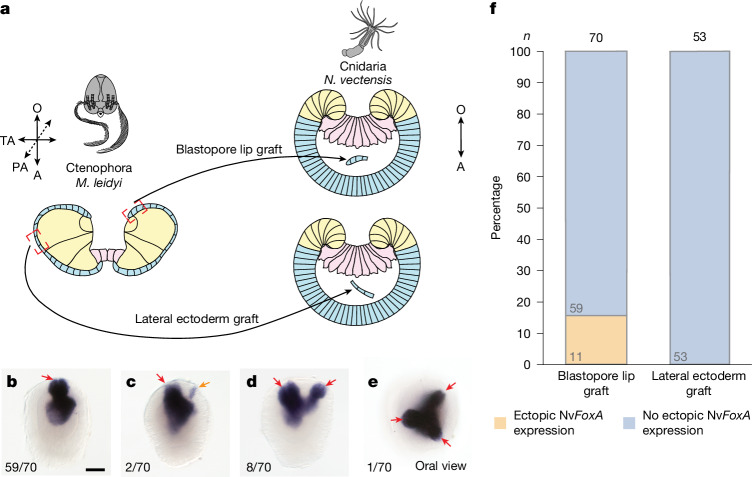
The ctenophore embryonic organizer can induce secondary axis formation in a cnidarian embryo. **a**, Schematic of the xenotransplantation experiment for grafting *M. leidyi* gastrula tissues into *N. vectensis* gastrula embryos. **b**–**e**, Images of phenotypes obtained in the xenotransplantation experiment, which analysed *N*. *vectensis* planula Nv*FoxA* expression after *M. leidyi* organizer xenotransplantation: no ectopic expression (**b**) or ectopic expression associated with no distinct pharynx (**c**), one morphologically distinct pharynx (**d**) or two morphologically distinct pharynxes (**e**). The expression of the pharyngeal marker gene Nv*FoxA* in *N. vectensis* embryos after transplantation of *M. leidyi* blastopore lip indicates the formation of multiple body axes. Red arrows indicate Nv*FoxA* expression associated with morphologically distinct mouth opening formation (**b**–**e**), whereas the orange arrow indicates ectopic expression without such association (**c**). Numbers in the bottom left present the fraction of embryos displaying this phenotype. Scale bar, 50 μm. **f**, Quantification of transplantation of the blastopore lip of *M. leidyi* gastrula, but not lateral ectoderm, can induce the ectopic expression of Nv*FoxA* in *N. vectensis* gastrula. Stacked bars show pooled proportions.

As our experiments confirmed that the ctenophore blastopore lip is an embryonic axial organizer, we investigated whether the *M. leidyi* blastopore lip can induce axis formation in other animals. We transplanted the blastopore lip from *M. leidyi* gastrula into the blastocoel of embryos of the sea anemone *N. vectensis* (Cnidaria) (Fig. 3a). We assessed the inductive capacity of the blastopore lip by analysing the expression of *N. vectensis FoxA* (Nv*FoxA*; throughout, the first two letters prefixing a gene or protein indicate the species), a pharyngeal marker, which is consistently associated with the formation of a secondary axis during induction in *N. vectensis*^2^. As *M. leidyi* lacks a *FoxA* orthologue^10^, the specific Nv*FoxA* probe used in the xenotransplantation assay can only detect Nv*FoxA*-expressing *N. vectensis* cells. Thus, an Nv*FoxA*-positive signal in the ectopic axis reflects the recruitment and pharyngeal specification of *N. vectensis* cells in the secondary axis induced by the ctenophore organizer. We observed ectopic Nv*FoxA* expression in 15.7% (11 out of 70) of the transplantations (Fig. 3b–f). No ectopic Nv*FoxA* expression was detected in any *N. vectensis* embryos grafted with *M. leidyi* lateral ectoderm (*n* = 53 total transplantations; Fig. 3f). Therefore, the embryonic organizer of ctenophores is capable of inducing an ectopic axis in a cnidarian, despite the deep evolutionary divergence of these two animal lineages. Therefore, similar mechanisms responsible for embryonic induction in Ctenophora must also be present in Cnidaria.

A reciprocal *N. vectensis* to *M. leidyi* blastopore lip transplantation would strengthen our argument that the molecular basis of axis induction is shared across Metazoa. However, such an experiment is not feasible for two reasons. First, embryonic cells of *M. leidyi* and *N. vectensis* show essentially no adhesion to each other. Second, *M. leidyi* embryos do not form a blastocoel at any stage of embryonic development that would enable stable retention of a heterologous donor graft. By contrast, the presence of a blastocoel in *N. vectensis* embryos allows stable retention of donor tissue transplanted from *M. leidyi*.

## β-Catenin and TGFβ specify the organizer

To characterize the inductive nature of the ctenophore organizer, we investigated potential signalling pathways that are involved in the organizer region in *M. leidyi*. β-Catenin signalling influences primary axis polarity both in bilaterian animals^11^ and in non-bilaterian animals (for example, *N. vectensis*, *Clytia hemisphaerica* and *Dynamena pumila*)^3,12,13^. Therefore, its role in axial patterning probably predates the emergence of bilaterally symmetric animals. We hypothesized that the β-catenin pathway represents a universal signalling pathway that underlies the function of the embryonic organizer in all multicellular animals, including ctenophores.

**Extended Data Fig. 4:**
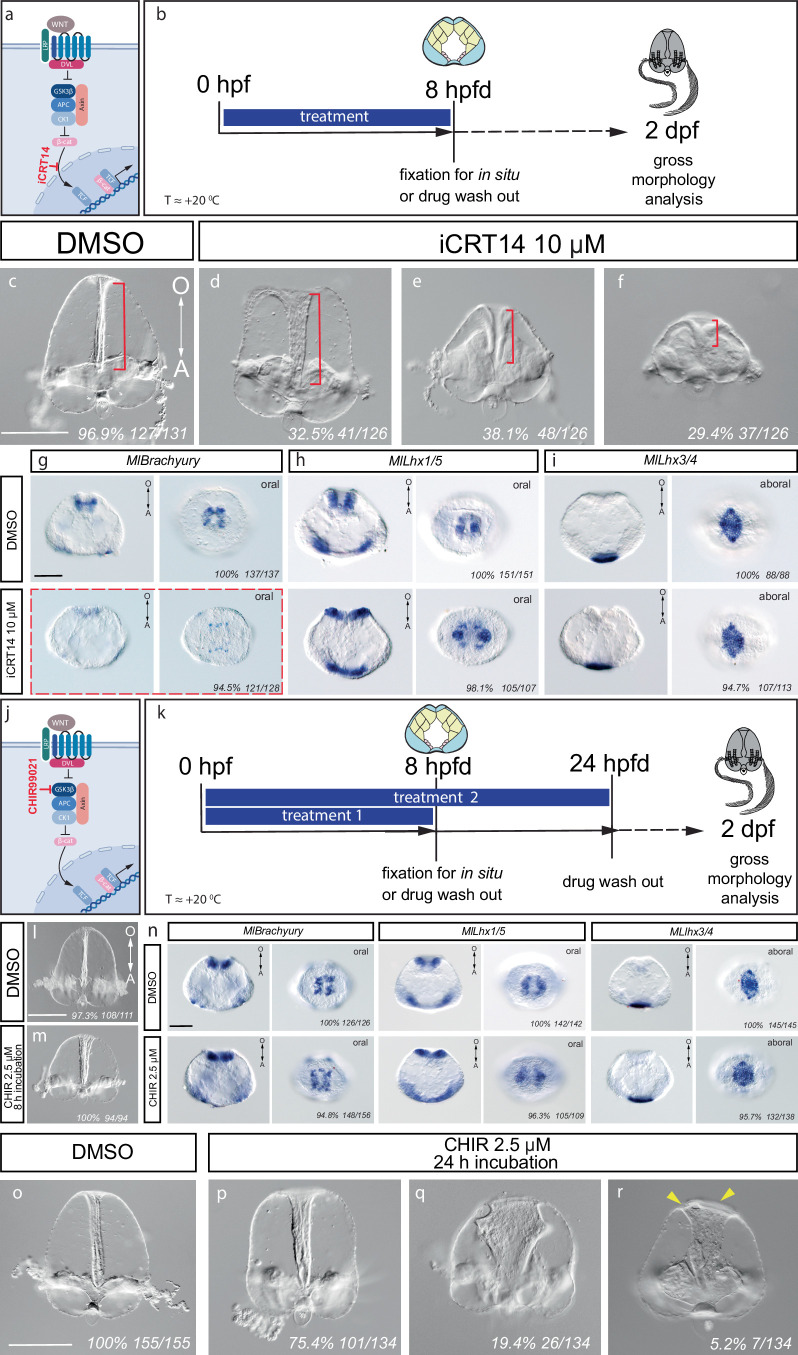
Effects of β-catenin signaling perturbation on ctenophore organizer development and specification. **a**–**i**, β-catenin signaling inhibition with iCRT14 affects the development and specification of the ctenophore organizer region. **a**, Scheme of Wnt/β-catenin signaling inhibition by iCRT14. **b**, Scheme of the experiment on β-catenin signaling inhibition by iCRT14. **c–f**, iCRT14 treatment inhibits pharynx elongation in *M. leidyi*. The red bracket marks the length of the pharynx. Scale bar, 250 μm. **g–i**, Expression of *MlBrachyury*, *MlLhx1/5*, and *MlLhx3/4* after inhibition of β-catenin signaling with iCRT14. *MlBrachyury* expression is strongly reduced (outlined by the red dashed box) (**g**), whereas expression of *MlLhx1/5* (**h**) and the aboral marker *MlLhx3/4* (**i**) remains unchanged. Scale bar, 40 μm. **j**–**r**, Early activation of β-catenin signaling with CHIR99021 does not affect the development and specification of the ctenophore organizer region. **j**, Scheme of Wnt/β-catenin signaling activation by CHIR99021. **k**, Scheme of the experiment on β-catenin signaling activation by CHIR99021. **l**,**m**, CHIR99021 treatment from 2 cells to 8 hpfd does not affect *M. leidyi* cydippid formation. Scale bar, 250 μm. **n**, Treatment with CHIR99021 from 2 cells to 8 hpfd does not affect expression patterns of *MlBrachyury*, *MlLhx1/5* and *MlLhx3/4*. Scale bar, 40 μm. **o–r**, CHIR99021 treatment from 2 cells to 24 hpfd leads to enlarged pharynx formation (**q**) or even to the formation of two mouths (**r**). Scale bar, 250 μm. The numbers in the bottom of images show the fractions of embryos demonstrating this phenotype. hpf, hours post-fertilization; hpfd, hours post first division; dpf, days post-fertilization.

To determine the contribution of β-catenin signalling to the organizer and oral structure development in *M. leidyi*, we treated embryos with the β-catenin pathway inhibitor iCRT14 and the activator CHIR99021. Embryos treated with 10 μM iCRT14 from the 2–4-cell stage to the end of gastrulation (8 h after the first division) (Extended Data Fig. 4a,b) developed into cydippid larvae, of which 67.5% (85 out of 126) exhibited a shortened pharynx and tentacle defects (Extended Data Fig. 4e,f). This phenotype closely recapitulated that observed in embryos of *M. leidyi* with *Brachyury* (Ml*Brachyury*) knocked down or knocked out^14,15^. Inhibition of β-catenin signalling with iCRT14 also strongly reduced Ml*Brachyury* expression (Extended Data Fig. 4g). In normal development, *Brachyury* is expressed during gastrulation in ectodermal cells surrounding the blastopore and continues to be expressed in the developing pharynx^16^. In *M. leidyi*, expression of the ancestral organizer-associated gene Ml*Lhx1/5*, as well as the aboral marker Ml*Lhx3/4* (ref. ^17^), remained unchanged (Extended Data Fig. 4h,i).

Treatment with the β-catenin activator CHIR99021 (2.5 μM) from the 2–4-cell stage to 8 h after the first division (Extended Data Fig. 4j,k) did not lead to any visible morphological changes (Extended Data Fig. 4l,m), nor did it alter the expression of Ml*Brachyury*, Ml*Lhx1/5* or Ml*Lhx3/4* (Extended Data Fig. 4n). Extending the treatment to 24 h after the first division, however, resulted in clear phenotypic effects by 2 days after fertilization (Extended Data Fig. 4o–r). Specifically, 19.4% of larvae (26 out of 134) developed a markedly expanded pharynx (Extended Data Fig. 4q), and 5.2% (7 out of 134) formed two closely adjacent mouth openings (Extended Data Fig. 4r). In the latter cases, the two mouths were positioned close to each other, which was probably due to the separation of the abnormally expanded pharynx by prolonged β-catenin activation. Increasing the concentration of CHIR99021 or prolonging exposure did not enhance these phenotypes but instead caused developmental arrest and embryo death. Previous work similarly reported minimal effects on early *M. leidyi* development after β-catenin signalling activation using compounds such as LiCl or alsterpaullone^18^. The fact that blocking β-catenin signalling in *M. leidyi* inhibits Ml*Brachyury* expression, whereas activation of the pathway at early developmental stages has no detectable effect, suggests that β-catenin is necessary for Ml*Brachyury* expression but not sufficient to activate expression or expand the Ml*Brachyury*-positive domain.

**Extended Data Fig. 5:**
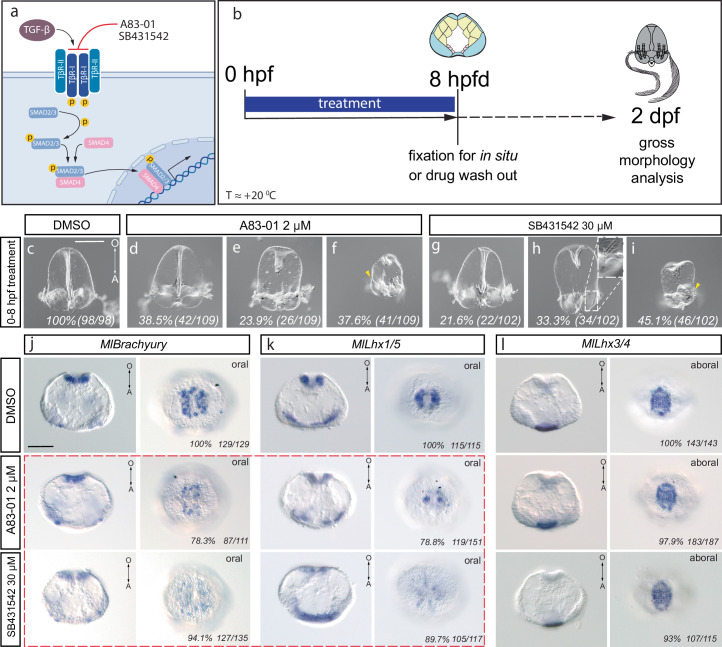
TGFβ-Smad2/3 signaling inhibition with A83-01 and SB431542 affects the development and specification of the ctenophore organizer region. **a**, Scheme of TGFβ-Smad2/3 signaling inhibition by A83-01 and SB431542. **b**, Scheme of the experiment on TGFβ-Smad2/3 signaling inhibition by A83-01 and SB431542. **c–i**, A83-01 and SB431542 treatment from 2 cells to 8 hpfd inhibits pharynx formation in *M. leidyi* cydippid (**e,f,h,i**). Scale bar, 250 μm. Yellow arrowheads point to well-developed comb row cilia. The boxed inset shows well-developed comb row cilia in a different focal plane. Inhibition of TGFβ-Smad2/3 also leads to defects in tentacle bulb development (**f, h, i**). Scale bar: 250 μm. **j–l**, Expression of *MlBrachyury*, *MlLhx1/5*, and *MlLhx3/4* after inhibition of TGFβ–Smad2/3 signaling with A83-01 and SB431542. Expression of *MlBrachyury* and *MlLhx1/5* is strongly reduced (outlined by red dashed box) (**j**, **k**), whereas expression of the aboral marker *MlLhx3/4* remains unchanged (**l**). Scale bar, 40 μm. The numbers in the bottom of images show the fractions of embryos demonstrating this phenotype. hpf, hours post-fertilization; hpfd, hours post first division; dpf, days post-fertilization.

It has previously been demonstrated that blocking the TGFβ–SMAD2/3 signalling cascade with SB431542, an inhibitor of the TGFβ type I receptor (ALK4/5/7), disrupts development of the ctenophore organizer region, leading to pharyngeal malformation^19^. On the basis of these findings, we proposed that TGFβ–SMAD2/3 signalling may contribute to organizer formation and/or organizer-mediated induction in *M. leidyi*. All identified receptors of the TGFβ family in *M. leidyi* share the canonical receptor architecture: an extracellular domain, a single-pass transmembrane segment and an intracellular serine–threonine kinase domain. Moreover, the three type I receptors (encoded by Ml*TgfrIa*–Ml*TgfrIc*) contain a glycine–serine repeat region immediately adjacent to the kinase domain, a characteristic consistent with their classification as type I (ALK) receptors^20^. Phylogenetic analyses do not clearly separate these receptors into BMP, Activin or Nodal subclasses. Therefore, we refer to them here simply as TGFβ type I and type II receptors^19^. Accordingly, to assess the role of TGFβ type I receptor activity in *M. leidyi* organizer formation and oral structure development, we treated embryos with two independent TGFβ receptor type I inhibitors, A83-01 and SB431542. Inhibition of TGFβ–SMAD2/3 signalling led to pharyngeal malformations^19^ (Extended Data Fig. 5a–i) and the downregulation of Ml*Brachyury* and another organizer-associated gene, Ml*Lhx1/5*, in the developing mouth of *M. leidyi*. By contrast, expression of the aboral marker Ml*Lhx3/4* remained unchanged (Extended Data Fig. 5j–l). In vertebrates, the *Lhx1/5* homologue *LHX1* (also known as *LIM1*) functions as an organizer-associated gene for which expression depends on TGFβ signalling mediated by SMAD2 and SMAD3 (TGFβ–SMAD2/3 signalling), and is specifically activated by Nodal and/or Activin ligands^21,22^. By contrast, in bilaterians, *Brachyury* expression typically depends on both TGFβ–SMAD2/3 and β-catenin signalling^23,24^. This characteristic is consistent with the knowledge that coordinated β-catenin–TGFβ–SMAD2/3 regulatory interactions have deep evolutionary roots and were already evident in pre-bilaterian lineages such as cnidarians (for example, *Hydra*)^25^.

**Extended Data Fig. 6:**
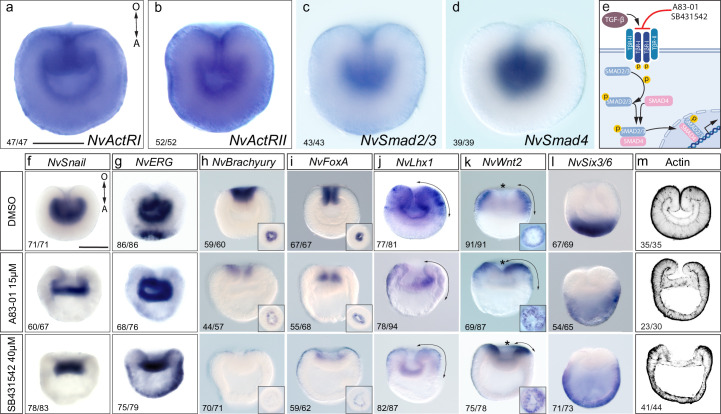
TGFβ-Smad2/3 signaling inhibition with A83-01 and SB431542 affects specification of the *N. vectensis* organizer region and oral-aboral patterning. **a–d**, Expression patterns of core TGFβ-Smad2/3 pathway components, *NvActRI* (**a**), *NvActRII* (**b**), *NvSmad2/3* (**c**) and *NvSmad4* (**d**). **e**, Scheme of TGFβ-Smad2/3 signaling inhibition by A83-01 and SB431542. **f–m**, Effects of A83-01 and SB431542 treatment. Expression of the endodermal marker *NvSnail* (**f**) is not affected. The endodermal domain of *NvERG* (**g**) remains unchanged, whereas its aboral domain expands towards the oral pole. Expression of *NvBrachyury* (**h**), *NvFoxA* (**i**) and *NvLhx1* (**j**) is downregulated. The *NvWnt2* expression domain shifts towards the blastopore (indicated by an asterisk) (**k**), and the aboral marker *NvSix3/6* expands towards the oral pole (**l**). The double arrow indicates the area of gene expression (**j**, **k**). **m**, Phalloidin staining of the 30 hpf gastrula upon A83-01 and SB431542 treatment. All embryos are fixed at 30 hpf. The image insets in the bottom right corners show the oral view of the embryo. The numbers in the bottom left corners show the fractions of embryos demonstrating this phenotype. Oral pole is up. Scale bar, 100 μm. O – oral, A – aboral.

To evaluate whether TGFβ–SMAD2/3 signalling may act during gastrulation in *N. vectensis*, we analysed the expression patterns of core pathway components, including the receptors Nv*ActRI* and *NvActRII* and the effector molecules Nv*Smad2/3* and Nv*Smad4*. Both receptors were expressed uniformly throughout the embryo at the gastrula stage (Extended Data Fig. 6a,b). Nv*Smad2/3* transcripts were also detected across the embryo, with increased levels in the endoderm (Extended Data Fig. 6c), whereas Nv*Smad4* transcripts were predominantly enriched in the endoderm (Extended Data Fig. 6d). Nv*Smad4* mRNA is maternally supplied^26^, which indicates that Nv*Smad4* transcripts are present in the zygote and may therefore support broader protein availability at later stages, including gastrulation. However, protein distribution cannot be directly inferred from mRNA data alone. Together, these observations suggest that the core components of the TGFβ–SMAD2/3 pathway are probably present during gastrulation and are available to support signalling activity in *N. vectensis*.

With respect to upstream signals, the *N. vectensis* genome seems to contain only two plausible SMAD2/3 branch ligands, Nv*Activin* and Nv*Gdf8* (which encodes myostatin)^27^. However, expression datasets of *N. vectensis* development have identified Nv*Activin* as the only one expressed during gastrulation^28,29^. Moreover, Nv*Activin* transcripts are found in the endoderm and become enriched towards the oral pole at this stage^29^. Developmental profiling has shown that Nv*Activin* is not maternally supplied but is instead zygotically activated at the blastula stage, immediately before the onset of gastrulation, and its expression is reported in the animal hemisphere, the region where gastrulation initiates^26^. Moreover, Nv*Activin* expression is upregulated following treatment with the GSK3β inhibitor 1-azakenpaullone, which activates canonical β-catenin signalling. This result raises the possibility that β-catenin activity contributes to the regulation of Nv*Activin* expression during early *N. vectensis* development^26^.

To investigate how TGFβ–SMAD2/3 signalling contributes to the formation of the organizer region in *N. vectensis*, we treated embryos with the TGFβ type I receptor inhibitors SB431542 and A83-01 (Extended Data Fig. 6e). Blockade of TGFβ–SMAD2/3 signalling did not affect expression of the endodermal marker Nv*Snail* (Extended Data Fig. 6f). Likewise, the endodermal expression domain of Nv*Erg* remained unchanged, although its aboral expression domain expanded across the ectoderm (Extended Data Fig. 6g). By contrast, inhibition of TGFβ–SMAD2/3 signalling resulted in downregulation of the blastopore-associated genes Nv*Brachyury* and Nv*FoxA*, as well as reduced expression of Nv*Lhx1* (Extended Data Fig. 6h–j). In *N. vectensis*, Nv*Lhx1* is not restricted to the organizer region but is broadly expressed across the oral hemisphere. Nv*Wnt2*, which is typically expressed in an equatorial ectodermal ring, expanded towards the blastopore margin following A83-01 treatment. By contrast, after SB431542 treatment, Nv*Wnt2* expression disappeared from the equatorial region and was restricted to the blastoporal area (Extended Data Fig. 6k). The aboral marker Nv*Six3/6* expanded throughout the ectoderm (Extended Data Fig. 6l). The observed unaltered expression of endodermal markers after treatment supports a specific patterning effect and argues against nonspecific toxicity as the cause of reduced Nv*Brachyury* and Nv*FoxA* expression. Furthermore, gastrulation movements were disrupted (Extended Data Fig. 6m). The phenotype observed after TGFβ–SMAD2/3 signalling inhibition resembles the effect of WNT–β-catenin signalling suppression in *N. vectensis*. In both cases, the expression of blastopore lip-associated genes were reduced, midbody markers showed blastoporal expression and aboral markers expanded towards the oral end. These observations suggest that TGFβ–SMAD2/3 signalling and β-catenin act together in a shared regulatory network to control organizer formation and oral–aboral patterning^30,31^. Taken together, the present results make functional coupling between TGFβ–SMAD2/3 and β-catenin signalling in the *N. vectensis* blastoporal organizer the most plausible interpretation.

In bilaterians, TGFβ–SMAD2/3 signalling has a fundamental role in establishing primary axial patterning in sea urchins^32^ and it is essential for the formation of the embryonic organizer in chordates^33,34^. Together with our findings in ctenophores and cnidarians, this result supports the idea that alongside β-catenin, TGFβ–SMAD2/3 is a deeply conserved component of the gene regulatory networks that pattern the primary body axis and specify organizer activity.

## Induction is driven by β-catenin and TGFβ

**Extended Data Fig. 7:**
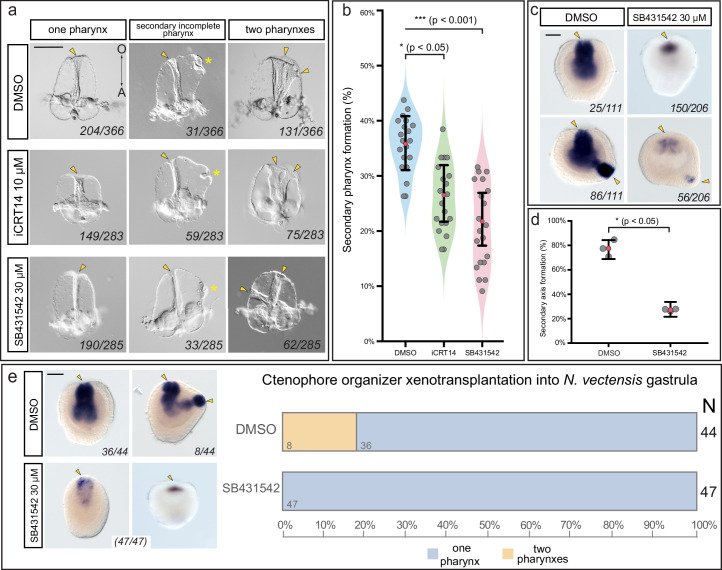
Signaling requirements for organizer-driven induction of secondary oral structures in *M. leidyi* and *N. vectensis* transplantation assays. **a**, Phenotypes obtained after transplanting the *M. leidyi* organizer into *M. leidyi* gastrulae treated with the β-catenin inhibitor iCRT14 or the TGFβ–Smad2/3 inhibitor SB431542. Yellow arrowheads indicate the mouth with a fully formed pharynx in cydippid larvae. Yellow asterisks indicate formation of a mouth-like structure without a pharynx. Scale bar, 250 μm. **b**, iCRT14 and SB431542 significantly reduced the frequency of larvae with two pharynxes after organizer transplantation. Relative to DMSO controls, iCRT14 reduced secondary pharynx induction (OR = 0.65, 95% CI 0.46–0.91, p = 0.012), as did SB431542 (OR = 0.50, 95% CI 0.35–0.71, p = 1.14 × 10^−4^). n = 20 independent biological replicates. **c**, Phenotypes obtained after transplanting the *N. vectensis* organizer into *N. vectensis* gastrulae treated with SB431542. Yellow arrowheads indicate *NvFoxA* expression in forming pharynxes. Scale bar, 50 μm. **d**, SB431542 significantly reduced the frequency of planulae with two pharynxes after organizer transplantation in *N. vectensis*. Relative to DMSO controls, SB431542 reduced ectopic NvFoxA expression (OR = 0.51, 95% CI 0.28–0.92, p = 0.028). n = 3 independent biological replicates. In **d** and **b**, individual grey points indicate replicate-level success proportions. Pink points indicate model-estimated probabilities (centre of error bars) from the binomial GLMM, and error bars represent 95% confidence intervals. Statistical significance was assessed using two-sided Wald z-tests from a binomial generalized linear mixed model (GLMM). No adjustments were made for multiple comparisons. **e**, SB431542 abolished induction in xenotransplantation experiments in which the *M. leidyi* organizer was transplanted into *N. vectensis* gastrulae. Yellow arrowheads indicate *NvFoxA* expression in forming pharynxes. Stacked bars show pooled proportions. Scale bar, 50 μm.

**Extended Data Fig. 8:**
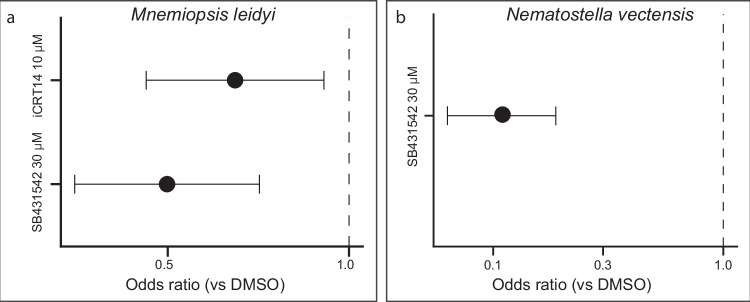
Effects of TGFβ-Smad2/3 and β-catenin inhibition on organizer-driven secondary oral structure induction in *M. leidyi* and *N. vectensis* transplantation assays. **a**, Forest plot showing the effects of SB431542 and iCRT14 on organizer-induced secondary pharynx formation in *M. leidyi* (n = 20 independent biological replicates per condition: DMSO control, SB431542 treatment, and iCRT14 treatment). **b**, Forest plot showing the effect of SB431542 on organizer-induced secondary pharynx formation in *N. vectensis* (n = 3 independent biological replicates per condition: DMSO control and SB431542 treatment). Points indicate odds ratios (centre of error bars) relative to DMSO controls; horizontal bars represent 95% confidence intervals. The dashed vertical line indicates no effect (OR = 1). Statistical significance was assessed using two-sided Wald z-tests from a binomial generalized linear mixed model (GLMM). No adjustments were made for multiple comparisons.

Since we found that β-catenin and TGFβ–SMAD2/3 signalling contribute to the development and specification of the embryonic organizer region in *M. leidyi* (Extended Data Figs. 4 and 5), and also discovered that the TGFβ–SMAD2/3 cascade is required for the development of the oral region and patterning of the primary body axis in *N. vectensis* (Extended Data Fig. 6e–m), we next investigated whether these signalling pathways are involved in the induction of ectopic oral structures following *M. leidyi* embryonic organizer grafting. We incubated grafted embryos for 12 h with the TGFβ–SMAD2/3 inhibitor SB431542 or the β-catenin signalling inhibitor iCRT14, using DMSO as a control. After washing out the inhibitors, embryos were scored at 2 days after fertilization. Treatment with SB431542 or iCRT14 significantly reduced organizer-induced secondary pharynx formation (Extended Data Figs. 7a,b and 8a). Complete secondary pharynx formation was used as the primary end point for organizer induction success.

As the involvement of TGFβ–SMAD2/3 signalling in embryonic induction in *N. vectensis* has not previously been examined (in contrast to β-catenin signalling^3^), we assessed its role using organizer transplantation assays. Transplantation of the *N. vectensis* embryonic organizer into host embryos resulted in induction that was suppressed after inhibition of TGFβ–SMAD2/3 signalling (Extended Data Figs. 7c,d and 8b), similar to the effects observed after transplantation of the *M. leidyi* organizer. Ectopic Nv*FoxA* expression was used as the primary end point for organizer induction success. Likewise, induction following xenotransplantation of the *M. leidyi* embryonic organizer into *N. vectensis* gastrulae was blocked by the TGFβ–SMAD2/3 inhibitor SB431542 (Extended Data Fig. 7e).

**Extended Data Fig. 9:**
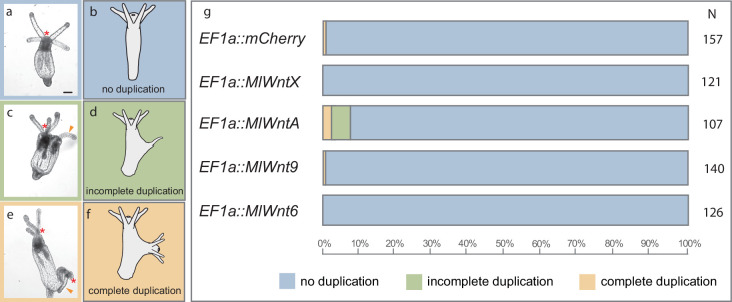
*M. leidyi* WntA induces a secondary axis when expressed in a *N. vectensis* embryo. **a–f**, Phenotypes observed in Wnt ligand expression experiments. Red asterisks indicate mouth openings, orange arrowheads indicate ectopic tentacles. **g**, Mosaic overexpression under *EF1α* promoter of only *M. leidyi MlWntA* is capable of inducing ectopic axes in the sea anemone *N. vectensis*. *N. vectensis* embryos were injected into single blastomeres at the 8–16 cell stage. One embryo with axis duplication in the mCherry-injected group most likely represents a natural spontaneous axis duplication. Scale bar, 100 μm.

Together, these findings indicate that organizer-mediated induction in *M. leidyi* requires both TGFβ–SMAD2/3 and β-catenin signalling. Organizer function is mediated by secreted extracellular ligands, whereas β-catenin acts as an intracellular effector. This result raises the question of whether WNT ligands contribute to organizer-mediated induction in *M. leidyi*. The *M. leidyi* genome contains four *Wnt* genes^10,35^. However, none seem to be expressed in the gastrula blastopore or later in the mouth region^35^. We therefore assumed that transcripts of WNT ligand might be present at earlier stages of development but were not identified using in situ hybridization. RNA sequencing (RNA-seq) data analysis has revealed the presence of transcripts for two WNT ligands, Ml*WntX* and Ml*Wnt6*, at pre-gastrulation stages^36,37^. However, functional experiments showed that mosaic overexpression of only Ml*WntA* was capable of inducing ectopic axes in the sea anemone *N. vectensis* (8.4% of cases, 9 out of 107) (Extended Data Fig. 9). Notably, both in situ hybridization and RNA-seq data have indicated that Ml*WntA* is expressed only later in development, after gastrulation^35–37^, and far from the organizer region^35^. Therefore, WNT ligands are unlikely to be responsible for providing the inductive properties at the *M. leidyi* blastopore. Instead, maternal β-catenin may drive early signalling events in the embryo in the absence of WNT ligand signals, similar to other systems (for example, zebrafish and *Xenopus*). That is, maternal β-catenin is activated during early embryonic development in a manner independent of WNT ligands and receptors^34,38^. Moreover, in *N. vectensis* embryos, active maternal β-catenin signalling is detected in regions where no WNT ligands are known to be expressed^39^.

## TGFβ ligands induce oral structures

The expression patterns of TGFβ signalling components during early *M. leidyi* development, including the gastrula stage, are consistent with a role for this pathway in mediating inductive signalling from the blastopore lip to surrounding tissues. The TGFβ-family ligands (ML368915a and ML102235a; here and throughout, the ‘ML’ prefix and associated numbers denote gene model identifiers from the *Mnemiopsis leidyi* genome portal^36,37^) are expressed in and around the blastopore^19^. Furthermore, in situ hybridization has shown that the type II receptor Ml*TgfRII* (ML08593b) and Ml*Smad2/3* (ML011743a) are expressed ubiquitously from early development to the cydippid stage^19^. Genome browser^36,37^ data also indicate that Ml*Smad4* (ML02191a), *TgfRIa* (ML082117a) and *TgfRIc* (ML046516a) mRNAs are maternally deposited in the egg. Together, these data suggest that essentially all cells are competent to respond to TGFβ signals. The localized production of TGFβ ligands at the blastopore probably provides the organizer with its inductive properties^19^.

**Fig. 4:**
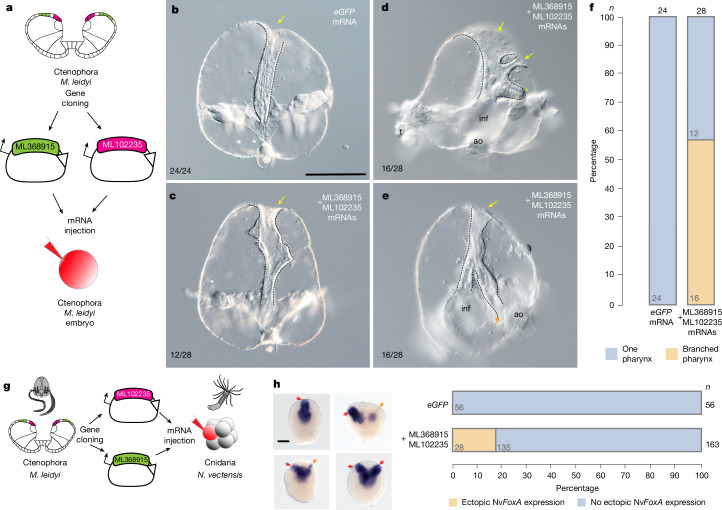
Overexpression of *M. leidyi* TGFβ-family ligands expressed in the blastoporal region is sufficient to induce pharyngeal branching and mouth duplication in both *M. leidyi* and *N. vectensis.* **a**, Schematic of the experiment. **b**, Injection of *eGFP* mRNA was used as a control and resulted in normal larvae with a single pharynx.** с**–**e**, Microinjection of RNAs encoding *M. leidyi* TGFβ-ML368915 (ML368915a) and TGFβ-ML102235 (ML102235a) into *M. leidyi* zygotes resulted in the development of larvae with either one pharynx (**c**) or pharynx branching and ectopic mouths (**d**,**e**). Arrows indicate mouth openings (**b**–**e**), and the asterisk (**d**) indicates pharyngeal diverticula without a mouth opening. The orange arrow (**e**) indicates pharynx branching with a mouth opening in another focal plane. Images are of embryos at 5 days after fertilization. Scale bar, 250 μm. **f**, Stacked bars showing pooled proportions of *M. leidyi* cydippids with the indicated phenotype. **g**, Schematic of the experiment for ectopic expression of *M.* *leidyi* TGFβ-ML368915 and TGFβ-ML102235 in a single *N. vectensis* blastomere at the 8-cell stage. **h**, Images (left) and quantification (right) of secondary body axis formation in *N. vectensis*, which either had no ectopic Nv*FoxA* expression (top left) or had ectopic Nv*FoxA* expression (top right and bottom left and right). Red arrowheads indicate Nv*FoxA* expression associated with pharynx development. The orange arrowheads indicate ectopic Nv*FoxA* expression in the absence of a morphologically developed pharynx. Scale bar, 50 μm. ao, aboral organ; inf, infundibulum.

Both *M. leidyi* and *N. vectensis* have the core components required for TGFβ–SMAD2/3 signalling at gastrula stages. Moreover, organizer-mediated induction is suppressed by TGFβ–SMAD2/3 inhibition. On the basis of these results, we hypothesized that the TGFβ-family ligands provide key extracellular input underlying organizer function in *M. leidyi*. To test this hypothesis, we injected a mixture of mRNAs encoding the *M. leidyi* TGFβ-family ligands TGFβ-ML368915 and TGFβ-ML102235 into *M. leidyi* zygotes (Fig. 4a). These ligands were selected because they are expressed at the oral pole near the blastopore in *M. leidyi* and are thought to participate in axial patterning of the *M. leidyi* embryo^19^. As a control, we injected *eGFP* mRNA, which did not cause detectable morphological changes in eGFP-injected embryos (24 out of 24; Fig. 4b). By contrast, overexpression of the TGFβ-family ligands led to pharyngeal branching and the formation of ectopic mouths in 57% of cases (16 out of 28; Fig. 4c–f). eGFP fluorescence was detectable by around 8 h after fertilization, which indicated that there was proper protein translation after mRNA injection, before the onset of cydippid formation (Supplementary Fig. 1). Successful protein production following mRNA microinjection in *M. leidyi* zygotes has been previously demonstrated^40^.

We next tested whether overexpression of these *M. leidyi* TGFβ-family ligands, TGFβ-ML368915 and TGFβ-ML102235, is sufficient to induce the formation of an ectopic pharynx in *N. vectensis*. mRNAs encoding these ligands were injected into individual blastomeres of 8-cell-stage *N. vectensis* embryos^3^ (Fig. 4g). Ectopic Nv*FoxA* expression was observed in 17% (28 out of 163) of embryos, whereas no ectopic Nv*FoxA* expression was detected following e*GFP* mRNA injection (*n* = 56; Fig. 4h).

Overexpression of the *M. leidyi* TGFβ ligand TGFβ-ML218835, which is expressed aborally during gastrulation, did not induce the formation of ectopic oral structures in either *M. leidyi* or *N. vectensis* (Supplementary Fig. 2). Microinjection of *M. leidyi* mRNA encoding TGFβ-ML218835 into *M. leidyi* zygotes delayed pharynx–infundibulum fusion in cydippid larvae (Supplementary Fig. 2e,f), even though TGFβ-ML218835 is expressed in the pharynx at the cydippid stage^19^. In *N. vectensis*, ectopic expression of *M. leidyi* TGFβ-ML218835 in a single blastomere at the 8-cell stage suppressed Nv*FoxA* expression (Supplementary Fig. 2g–m). This result raises the possibility that TGFβ-ML218835 may act more like a BMP-type ligand, consistent with observations in sea urchins in which BMP ligand overexpression similarly suppresses *FoxA* expression^41^.

Together, these findings indicate that the TGFβ-family ligands expressed around the *M. leidyi* blastopore are sufficient to trigger ectopic oral structure formation in both *M. leidyi* and *N. vectensis* embryos.

## Conclusion

**Fig. 5:**
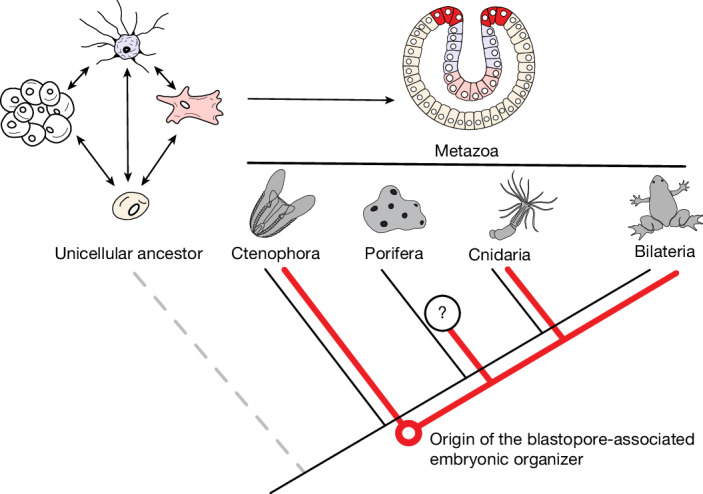
A proposed scenario for the emergence of the embryonic organizer in the common ancestor of all metazoans. Arrow show the transition from temporal cell differentiation in unicellular ancestors to spatial cell differentiation in multicellular embryos.

**Extended Data Fig. 10:**
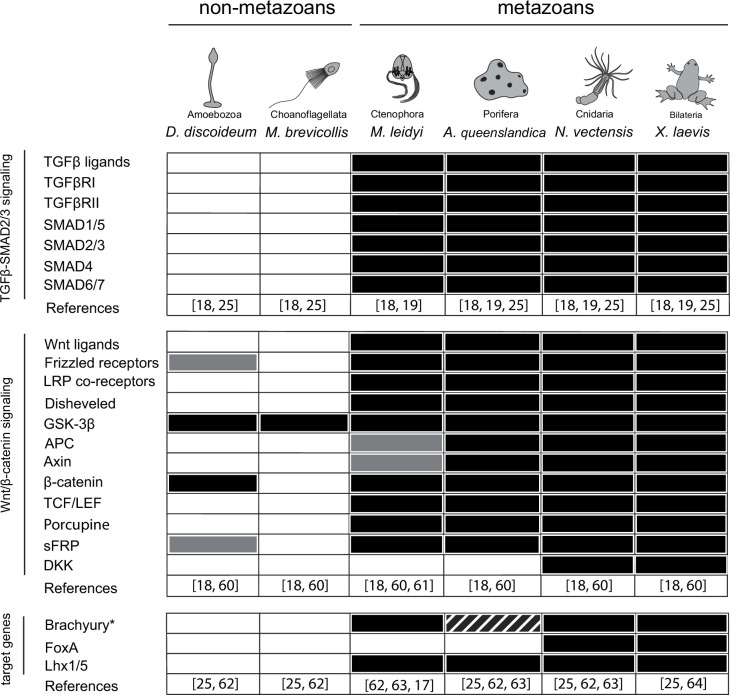
TGFβ and Wnt/β-catenin signaling components across unikont lineages. Columns - species. Rows - gene members involved in TGFβ and Wnt/β-catenin signaling, and target genes of these pathways. White boxes - no homologue reported in the literature. Grey boxes - putative/degenerate homologue. Black boxes - homologue present. Crosshatched boxes - no Brachyury homologue detected in *Amphimedon queenslandica*, but its homologue is present in other Porifera species (e.g. *Sycon ciliatum*). Asterisks next to Brachyury indicate that, although no homologue is detected in choanoflagellates or amoebozoans, a Brachyury homologue is present in other non-metazoans (e.g. ichthyosporeans and filastereans)^17–19,25,60–64^.

Our study provides a perspective on the evolution of the embryonic organizer in Metazoa and the emergence of multicellularity, and highlights the critical role of cell–cell communication in this process. The transition from unicellular to multicellular animal life required several key conditions to be met, including the evolution of mechanisms for cell–cell adhesion, diverse cell types temporarily capable of performing different functions and the organization of these cells into specific spatial patterns^42,43^. Although the basic genetic machinery for cell adhesion and specialization existed before metazoans^44,45^, it was the rise of signalling pathways for cell–cell communication that drove the evolution of complex multicellularity in animals^19^. TGFβ signalling is a clear example of this innovation^18,46^. A full set of TGFβ signalling components was identified in ctenophores^19^ (Extended Data Fig. 10), which indicated their role in the early stages of multicellularity. None of these components have been found in non-metazoan unikonts^44,45^. Based on our data, TGFβ cell–cell communication seems to be essential for embryonic induction in ctenophores. Our findings indicate that the emergence of this signalling pathway probably played a key part in the origin of the embryonic organizer during the transition to complex multicellularity (Fig. 5).

We showed that the *M. leidyi* blastopore lip shares key organizer properties with blastopore-associated organizers described in cnidarians and vertebrates. Across these lineages, blastopore-associated organizers seem to use a common regulatory logic in which Brachyury links upstream signalling to morphogenetic programs. In *M. leidyi*, Ml*Brachyury* is expressed at the blastopore lip and in the stomodeal and pharyngeal epithelium^16^, and knockout of Ml*Brachyury* blocks stomodeal invagination and disrupts pharynx morphogenesis^15^. Our data further suggest that both β-catenin and TGFβ–SMAD2/3 signalling act upstream to Ml*Brachyury* function in this region. Similar blastopore-associated roles have been described in *N. vectensis*, in which *Nv*Brachyury encircles the blastopore and is required for pharynx formation^47^ in a WNT-dependent axial network^48^. Also in vertebrates, *Brachyury* is induced by WNT–β-catenin and Nodal/Activin–SMAD2/3 and controls gastrulation morphogenesis^49^. In the filasterean *Capsaspora owczarzaki*, Co*Brachyury* is activated during life-cycle stages characterized by intense cell rearrangements^50^. This result is consistent with the idea that Brachyury regulated morphogenetic cell movements before the origin of animals and was later co-opted for cell migration and gastrulation morphogenesis^50^. Notably, cross-species assays in *X. laevis* support a conserved role for *M. leidyi* Ml*Brachyury* in controlling gastrulation morphogenesis^14^ and showed that *C. owczarzaki* Co*Brachyury* retains functional activity in *Xenopus* rescue assays^51^. Together, these findings support the view that Brachyury represents an ancient module that links upstream signalling to morphogenetic programs that has been retained across animal evolution and incorporated into blastopore-associated organizer networks. In this framework, the *M. leidyi* organizer can be seen as one of the earliest evolutionary realizations of an ancient Brachyury module that has become wired to both β-catenin and TGFβ signalling at the blastopore lip.

Our results indicate that organizer function in *M. leidyi* relies on a conserved β-catenin–TGFβ–SMAD2/3 signalling logic that has also been implicated in axis-inducing centres in other animals. In *Hydra*, a β-catenin–TGFβ–SMAD2/3 module specifies lateral budding sites and generates a new axis orthogonal to the primary body axis^25^. In vertebrates, including human stem-cell-derived organizers, β-catenin together with Activin and Nodal and downstream SMAD2/3 signalling defines organizers capable of inducing secondary body axes^52^. For example, in human gastruloids, these inputs are sufficient to self-organize an inducing centre that can generate a secondary body axis after grafting into chick embryos^52^. Placed in this context, the *M. leidyi* organizer extends the deployment of β-catenin–TGFβ–SMAD2/3 circuitry to ctenophores. This finding supports the idea that the core signalling cassette was already present early in animal evolution and was later adapted to control distinct modes of axis formation in different lineages.

Thus, our work links the earliest known evolutionary appearance of organizer activity to a conserved cell–cell signalling logic that could have facilitated the transition to spatial patterning in the embryos of the first animals.

## Methods

### Animal culture

A closed life cycle of *M. leidyi* based on cydippid reproduction has been established in the laboratory. The animals used to establish the culture were originally collected at the Kristineberg Marine Research Station.

Twenty to 40 individuals were maintained in 3-l Kreisel tanks at 17–19 °C in 27.5 ppt artificial seawater (ASW) (Red Sea Salt, Red Sea Fish Pharm), under a 19–5 light–dark cycle and fed once daily with *Brachionus plicatilis* (Rotifera). Feeding was performed manually using a Pasteur pipette by adding 3 drops of a concentrated rotifer suspension (100,000–150,000 rotifers per ml) per 3-l tank. Water in the Kreisel tanks was completely changed manually once every 2 weeks. Rotifers were fed with a commercial concentrated microalgae solution (RGcomplete, Reef Nutrition) and cultured according to a previously described protocol^15^.

For embryo collection, 10–20 cydippids (0.5–0.8 cm in size) were transferred into 200–250 ml beakers before the dark period of the light–dark cycle. Spawning occurred synchronously about 2–2.25 h after the start of the next light cycle. More than 90% of embryos (up to about 99%) developed synchronously. A small fraction of embryos lagged behind by one cleavage division at early stages; however, this difference was no longer detectable by the gastrula stage. After spawning, animals were returned to 3-l Kreisel tanks. Under these conditions, animals spawned daily.

*Nematostella vectensis* polyps were maintained in 16% ASW at 18 °C in the dark and fed daily *Artemia salina* nauplii. To induce spawning, the polyps were transferred to a 25 °C illuminated incubator for 10 h. Eggs were fertilized for 30 min, dejellied in 3% l-cysteine–ASW solution and then washed six times in ASW^53^.

No ethical approval was required for work with invertebrate species (*M. leidyi* and *N. vectensis*).

### Transplantation experiments

Specific parts of the donor gastrula (around 4 h after fertilization, at 17–19 °C, mid-gastrulation) were excised with a fine scalpel (Feather Sterile MicroScalpel 15 Deg, 72045-15, Feather Safety Razor) and transplanted into a host embryo at the same stage of development. The tissues were transplanted to the lateral side of the host embryo at the tentacular axis. A single incision (around 30 μm deep and 40 μm long) was made in the host embryo, and a donor explant was gently inserted into this slit so that its internal surface lay against the wound of the host embryo. The graft adhered immediately and became firmly attached to the host tissues within the next 5–7 min (Extended Data Fig. 1). Following grafting, embryos were incubated in plastic Petri dishes coated with 2% agarose. The agarose-coated bottom prevented embryo adhesion to the plastic surface and further mechanical damage of the embryos. The results of transplantation were analysed in cydippids at day 2  after fertilization.

To follow the distribution of the grafted tissues in the host animals, we labelled the embryos with the vital membrane dye FM4-64FX (F34653, Invitrogen) or FM1-43FX (F35355, Invitrogen). Before transplantation, the vitelline membrane was manually removed from embryos at the 2–2.5 h after fertilization stage. Embryos were then transferred to a staining solution for 1 h (ASW containing FM4-64FX (or FM1-43FX) at 10 μg ml^–1^) and incubated in the dark. After staining, embryos were rinsed three times in seawater to remove excess dye and maintained in seawater until the desired developmental stage. Tissue from stained with FM4-64FX embryos was then transplanted into unstained (or stained with FM1-43FX) embryos.

The same grafting methodology was used for xenotransplantation experiments. Specific parts of the donor *M. leidyi* gastrula were transplanted into the blastocoel of gastrulating *N. vectensis* embryos. These manipulations were performed in ASW with a salinity of 22–23%. For *N. vectensis* transplantations, all the manipulations were the same except that they were performed in 16% ASW. After grafting, *N. vectensis* embryos were incubated in plastic Petri dishes. The embryos were fixed at 58 h after fertilization.

### Pharmacological treatments

To inhibit TGFβ–SMAD2/3 signalling, embryos were treated with the ALK4/5/7 (TGFβ type I receptors) inhibitors A83-01 (HY-10432A, MedChemExpress) or SB431542 (S4317, Sigma-Aldrich). A83-01 was prepared by diluting a 10 mM stock solution (in DMSO) in ASW. SB431542 was prepared from a 50 mM stock solution (in DMSO) and diluted in ASW immediately before use. Final working concentrations were 2 µM A83-01 for *M. leidyi* and 15 µM for *N. vectensis*; SB431542 was used at 30 µM for *M. leidyi* and at 30 or 40 µM for *N. vectensis*, depending on the experiment. Control embryos were treated with an equal volume of DMSO. Treatments were initiated at the 2–4-cell stage in both species.

For inhibition of β-catenin signalling in *M. leidyi*, embryos were treated with 10 µM of the β-catenin inhibitor iCRT14 (SML0203, Sigma-Aldrich) from the 2–4-cell stage until the end of gastrulation (8 h after the first division). For β-catenin activation, embryos were treated with 2.5 µM CHIR99021 (SML1046, Sigma-Aldrich) either from the 2-cell stage to 8 h after the first division (early treatment) or, where indicated, extended to 24 h after fertilization (prolonged treatment).

For transplantation-based induction assays, embryos with grafted tissues were incubated for 12 h in inhibitor-containing ASW (SB431542 for TGFβ–SMAD2/3 inhibition or iCRT14 for β-catenin inhibition) with DMSO controls in parallel. Inhibitors were then washed out, and embryos were scored at 2 days after fertilization (*M. leidyi*) or fixed at the indicated time points (*N. vectensis*).

### In situ hybridization

The in situ hybridization protocol for *M. leidyi* was developed based on a published protocol for the sea anemone *N. vectensis*^54^. Tissues were first fixed in ice-cold 3.7% formaldehyde and 0.2% glutaraldehyde in PBS for 15 min on ice, followed by an additional 1 h in 3.7% formaldehyde in PBS at room temperature on a shaker. The fixative solution was replaced with PTw buffer (1× PBS (P4417, Sigma) and 0.1% Tween-20, pH 7.4), and the embryos were washed 3 times in PTw for 15 min at room temperature, followed by gradual transfer to 100% methanol and stored at −20 °C until use.

Digoxigenin-labelled RNA probes were synthesized using a MEGAscript SP6 Transcription kit (AM1330, Invitrogen). Fixed *M. leidyi* embryos were rehydrated through 60% and 30% methanol in PTw, digested with 80 μg ml^–1^ Proteinase K (AM2546, Thermo Fisher Scientific) in PTw for 40 s and then washed in 2 mg ml^–1^ glycine in PTw.

The DIG-labelled RNA probes, diluted to 0.5 ng ml^–1^ in hybridization solution, were incubated with the embryos overnight at 58 °C. Probe detection was performed using anti-Digoxigenin-AP Fab fragments (11093274910, Roche Diagnostics), diluted 1:2,000 in 0.5% blocking reagent (11096176001, Roche Diagnostics) in 1× MAB. This was followed by a substrate reaction using a mixture of NBT and BCIP (NBT, 11383213001; BCIP, 11383221001, Roche Diagnostics), as previously described^3^.

In situ hybridization with *N. vectensis* embryos was carried out following previously published protocols^3,54^.

Clones of Nv*FoxA* (GenBank: AY457634), Nv*Brachyury* (GenBank: AF540387), Nv*Six3/6* (GenBank: KC137590), *NvWnt2* (GenBank: AY725201), Nv*Snail* (GenBank: AY651960), Nv*Lhx1* (GenBank: BAH58087), Nv*Erg* (GenBank: EF427936), Ml*Brachyury* (GenBank: DQ988137.1), Ml*Lhx1/5* (GenBank: JF912807) and Ml*Lhx3/4* (GenBank: JF912808) were used to generate DIG-labelled RNA probes.

### mRNA microinjections

For overexpression experiments of *M. leidyi* TGFβ-family ligands in *N. vectensis*, full-length coding sequences of *M. leidyi* TGFβ-ML368915 (GenBank: JN380186), TGFβ-ML102235 (GenBank: JN380181.1), TGFβ-ML218835 (GenBank: JN380180) and control *eGFP* mRNA were cloned into pCRII vectors, flanked by the SP6 promoter and the SV40 polyA sequence. The full-length coding sequences of *M. leidyi* TGFβ ligands were amplified from *M. leidyi* cDNA using the following gene-specific primers: TGFβ-ML368915_dir GGCGCGCCAAAAAAATGCTTCACCTAGTTCTCGTTTTGTC; TGFβ-ML368915_rev CCTGCAGGTTATCGGCAGCTGCAGGAGTCGACAAC; TGFβ-ML102235_dir GGCGCGCCAAAAAAATGAGGACACTGAATTTGTTCCTAC; TGFβ-ML102235_rev CCTGCAGGTCACTTACAGCCACACTCTGTAAC; TGFβ-ML218835_dir GGCGCGCCAAAAAAATGGTTTGGCTGCTACTACTTTTATAC; and TGFβ-ML218835_rev CCTGCAGGTCACTCACAACCACACTGTTCGACC. The pCRII vectors containing the respective cDNAs were linearized with NotI, and mRNA was transcribed using a SP6 mMessage mMachine kit (AM1340, Invitrogen). Each mRNA were diluted to a final concentration of 0.35 µg µl^–1^. Fluorescent dextran-Alexa594 (D22913, Invitrogen) was co-injected as a tracer.

Microinjections into *M. leidyi* eggs were performed using a Pneumatic PicoPump microinjector (SYS-PV830, World Precision Instruments) equipped with a negative pressure function. Negative pressure (or suction pressure) was used to break the plasma membrane and allow the injection needle to enter the egg^55^. A Laboport N86 KT.18 pump was connected to the microinjector to generate negative pressure.

Microneedles (TW100F-4, World Precision Instruments) were pulled using a P-2000 puller (Sutter Instrument) with the following settings: heat, 260; filament, 4; velocity, 50; delay, 150; and pull, 150. The injection needle was loaded with mRNA solution from the end. The holding pipette was made from a Pasteur pipette with the tip rounded by heating and scraping with sandpaper^56^. For injection, a lid from a 4-well plate (176740, Nunc, Thermo Fisher Scientific) was placed under an inverted microscope (Axiovert 100, Carl Zeiss) and filled with ASW. Fertilized eggs were immobilized using a holding pipette by gentle suction. The injection needle was positioned horizontally and first passed through the outer vitelline membrane. After passing through the outer vitelline membrane, the needle tip was positioned at the plasma membrane. Negative pressure was then applied while the needle was gently moved into the egg. The injector was subsequently switched back to positive pressure, and a single injection pulse was applied (around 3–5 pl of the injection solution per egg) (Supplementary Video 1). Injected eggs were transferred to 35 mm Petri dishes containing ASW for further development.

### WNT ligand expression in *N. vectensis* embryos

For mosaic overexpression assays, the full-length coding sequences of *M. leidyi* *WntA* (GenBank: HM448813), *Wnt6* (GenBank: HM448814), *Wnt9* (GenBank: HM448815) and *WntX* (GenBank: HM448816) were cloned into pCRII-EF1α vectors (derived from pCRII-TOPO) under the control of the ubiquitous *N. vectensis* *Ef1a* promoter. The full-length coding sequences of *M. leidyi* WNT ligands were amplified from *M. leidyi* cDNA using the following gene-specific primers: MlAscWnt6F GGCGCGCCATGTCTGTCAGGGGAATCCTGTGC; MlSbfWnt6R CCTGCAGGCGCTGTCACGTACACCGAGTT; MlAscWntAF GGCGCGCCGCATATGCCTCCGCTGTTATTA; MlSbfWntAR CCTGCAGGGGTTAGCTATTGCAGGTGTAGGTG; MlAscWnt9F GGCGCGCCTGTAGATGGAGTTTCAGTCCCG; MlSbfWnt9R CCTGCAGGCGAGGCCTAATTGCAGTAATGA; MlAscWntXF GGCGCGCCCGCTCGCCTGAAATATATGG; and MlSbfWntXR CCTGCAGGCGCCTATAAAGTCCGGGAAT. Single blastomeres at the 8–16-cell stage were injected with a mixture containing 50 ng μl^–1^ plasmid DNA, 1× I-SceI buffer, 0.1 µg µl^–1^ fluorescent dextran-Alexa594 (D22913, Invitrogen) and 0.2 U μl^–1^ I-SceI meganuclease (New England Biolabs, R0694S). The mix was incubated for 30 min at 37 °C before injection^57^. As a control, we used the same pCRII-EF1α plasmid driving expression of the fluorescent reporter mCherry. After injection, embryos were cultured at 20 °C and raised to the primary polyp stage (7–9 days and fertilization), when phenotypes were scored.

### Phalloidin staining

For phalloidin staining, *N. vectensis* embryos were first fixed in a solution containing 4% paraformaldehyde and PTw (1× PBS, 0.1% Tween 20 and 0.2% Triton X-100) for 1 h at room temperature. Following fixation, they were washed five times with PTw. A staining solution was prepared by adding 2 µl of phalloidin-AlexaFluor488 1 µl ml^–1^ (A12379, Invitrogen) per 100 µl PTw, and the embryos were stained overnight at 4 °C. After three 15-min washes with PBS, the embryos were embedded in Vectashield (H-1000, Vector Laboratories).

### Microscopy

Embryos and cydippids were imaged using a fluorescence microscope (Carl Zeiss AxioImager.M2, Carl Zeiss Microscopy) equipped with a digital camera (pco.panda 4.2, Excelitas PCO). Images were acquired using a Plan-Apochromat ×20/0.8 objective with DIC and/or fluorescence microscopy. Samples were mounted under a coverslip supported by small modelling clay spacers at its four corners to prevent compression and allow immobilization. Live specimens were imaged in seawater. Nomarski and fluorescence images were overlaid in Fiji (v.2.14.0) software^58^.

Samples after in situ hybridization were imaged using either a Nikon Eclipse 80i microscope (Nikon) equipped with a Nikon DS-Fi1 camera (Nikon) and a Plan-Apochromat ×20/0.75 objective or an Axioscope 5 microscope (Carl Zeiss Microscopy) equipped with an Axiocam 503 colour camera and an EC Plan-Neofluar ×20/0.5 objective. Samples were mounted in 80% glycerol under a coverslip supported by small modelling clay spacers at its four corners to prevent compression. Slight movement of the coverslip allowed the embryo to be rotated under it, which enabled imaging from different angles.

Confocal imaging was performed using a Carl Zeiss LSM 980 microscope (Carl Zeiss Microscopy). For analysis of overall morphology, *M. leidyi* cydippids were fixed in 4% paraformaldehyde in PBS, stained with DAPI (1 µg ml^–1^) in PBS for 30 min at room temperature, gradually transferred to 80% glycerol and mounted under a coverslip supported by small modelling clay spacers at its four corners to prevent compression. *Nematostella vectensis* embryos stained with phalloidin were mounted and imaged in the same way. Live embryos and cydippids were mounted under a coverslip supported by modelling clay spacers in ASW. In some cases, cydippids were gently compressed to reduce movement during imaging. A C-Apochromat ×40/1.20 W objective was used for imaging *M. leidyi* embryos, whereas a Plan-Apochromat ×20/0.8 objective was used in all other cases.

### Statistical analysis

All statistical analyses were performed in R (v.4.6.0; R Foundation for Statistical Computing). Analyses were conducted on replicate-level embryo counts rather than percentages. Each biological replicate represents an independent cohort of embryos obtained from a separate spawn or collection, and embryos were scored once (no repeated measures). For organizer transplantation assays, embryos were classified according to phenotypic outcomes. Complete secondary pharynx formation (two pharynxes) was used as the primary end point for organizer induction success in *M. leidyi*, whereas ectopic secondary Nv*FoxA* expression was used as the primary end point in *N. vectensis*. For each biological replicate and treatment condition, the number of embryos meeting the defined end point (successes) and the total number of embryos scored were recorded, which produced binomial count data with replicate-specific denominators. Treatment effects were evaluated using a binomial generalized linear mixed model with a logit link, fitted to replicate-level counts, with treatment as a fixed-effect variable and biological replicate included as a random intercept to account for between-replicate variability (cbind(successes, failures) ~ treatment + (1 | replicate)). We validated that the model explained the data significantly better than the null model (cbind(successes, failures) ~ 1 + (1 | replicate)) with a two-way analysis of variance (*α* = 0.001). Effect sizes are reported as odds ratios (OR) relative to DMSO controls with 95% confidence intervals (CI) obtained from the model. Two-sided *P *values were derived from Wald *z*-tests for fixed effects, and significance was assessed at *α* = 0.05. Data processing and statistical modelling were performed using the packages readxl (v.1.4.5), dplyr (v.1.2.1), lme4 (v.2.0-1), broom.mixed (v.0.2.9.6) and emmeans (v.2.0.1). Microsoft Excel (Microsoft 365) was used for data handling and preliminary data organization.

No statistical methods were used to predetermine sample sizes. Experiments were not randomized because embryos were obtained from synchronous spawns and showed minimal variability before manipulation. Investigators were not blinded during data collection or outcome assessment because the analysed phenotypes were morphologically distinct.

### Experimental criteria and viability

Under our standard culture conditions, >90% (up to about 99%) of fertilized *M. leidyi* eggs developed into morphologically normal cydippids at 2 days after fertilization, and fertilized eggs reaching this stage were considered viable. Datasets were excluded if viability in the untreated or DMSO control group at 2 days after fertilization dropped below 90%. In transplantation experiments, survival of manipulated embryos was 90–100%. In pharmacological inhibition experiments, survival in inhibitor-treated embryos was slightly reduced relative to DMSO controls, but the difference never exceeded 5%.

### Figures and illustrations

Figures were prepared using Adobe Photoshop 2026 (v.27.0) and Adobe Illustrator 2026 (v.30.3).

### Reporting summary

Further information on research design is available in the Nature Portfolio Reporting Summary linked to this article.

## Online content

Any methods, additional references, Nature Portfolio reporting summaries, source data, extended data, supplementary information, acknowledgements, peer review information; details of author contributions and competing interests; and statements of data and code availability are available at 10.1038/s41586-026-10643-z.

## Supplementary information

Supplementary Information Supplementary Figs. 1 and 2.

Reporting Summary

Supplementary Video 1 Microinjection of *M. leidyi* zygotes. Fertilized eggs were immobilized using a holding pipette. The injection needle was inserted through the vitelline membrane, negative pressure was applied to penetrate the plasma membrane and a single injection pulse was applied.

## Data Availability

All data supporting the findings of this study are available in the Article, its Extended Data and Supplementary Information, as well as in external repositories as detailed below. The gene sequences used for the production of in situ hybridization probes and mRNA synthesis are available in the GenBank database (www.ncbi.nlm.nih.gov/genbank/) under the following accession numbers: AY457634, AF540387, KC137590, AY725201, AY651960, BAH58087, EF427936, DQ988137.1, JF912807, JF912808, JN380186, JN380181.1, JN380180, HM448813, HM448814, HM448815 and HM448816. Data on temporal gene expression during *M. leidyi* development can be accessed through the *M. leidyi* Genome Project Portal (https://research.nhgri.nih.gov/mnemiopsis/). Raw replicate-level counts used for statistical analysis are available in CodeOcean (10.24433/CO.6037194.v1)^59^.

## Code availability

All custom R scripts used for statistical analysis are available in a CodeOcean capsule (10.24433/CO.6037194.v1)^59^. The code is openly accessible and provided under an open-source licence. No restrictions apply to access or reuse.
