# Supplementary material for: A blastoporal organizer in a ctenophore
Source: Nature. 2026 Jun 17;655(8124):963–70. doi: 10.1038/s41586-026-10643-z (PMC13391361; doi:10.1038/s41586-026-10643-z)
Supplement: Supplementary file 1 — Supplementary Figs. 1 and 2. [file 41586_2026_10643_MOESM1_ESM.pdf]

nature portfolio

https://doi.org/10.1038/s41586-026-10643-z

Supplementary information

A blastoporal organizer in a ctenophore

In the format provided by the authors and unedited

Supplementary figures and legends

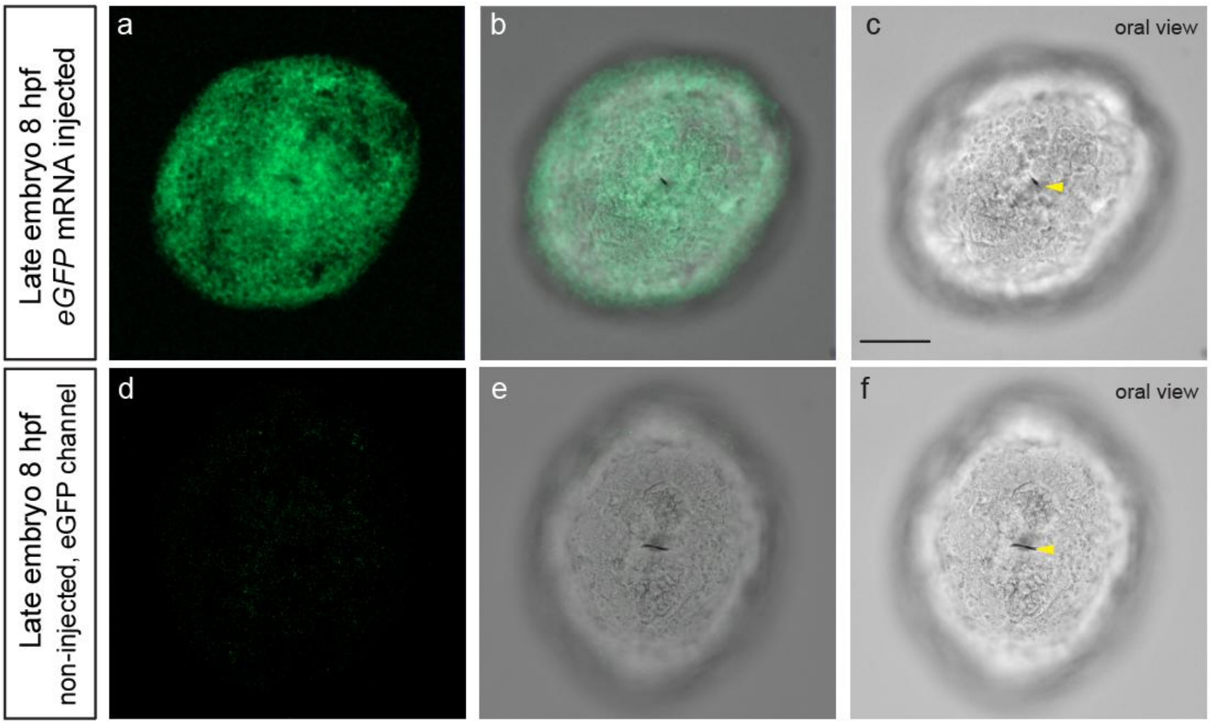

Figure S1: eGFP expression following mRNA microinjection in *Mnemiopsis leidyi* embryos. a–c, Embryos injected with *eGFP* mRNA at the zygote stage, imaged at ~8 hpf. **a**, eGFP fluorescence. **b**, Overlay of eGFP fluorescence and transmitted light. **c**, Transmitted light image. **d–f**, Uninjected control embryos at the same stage. **d**, eGFP channel showing no detectable signal. **e**, Overlay of eGFP channel and transmitted light. **f**, Transmitted light image. Fluorescence images in panels **a** and **d** represent confocal z-stack projections. All images were acquired using identical imaging settings (laser power, pinhole size, digital gain, and offset). For each condition (*eGFP* mRNA-injected and non-injected), imaging was performed on at least n = 3 individual embryos. Yellow arrowheads indicate the blastopore. Scale bar: **a–f**, 50 µm. hpf, hours post-fertilization.

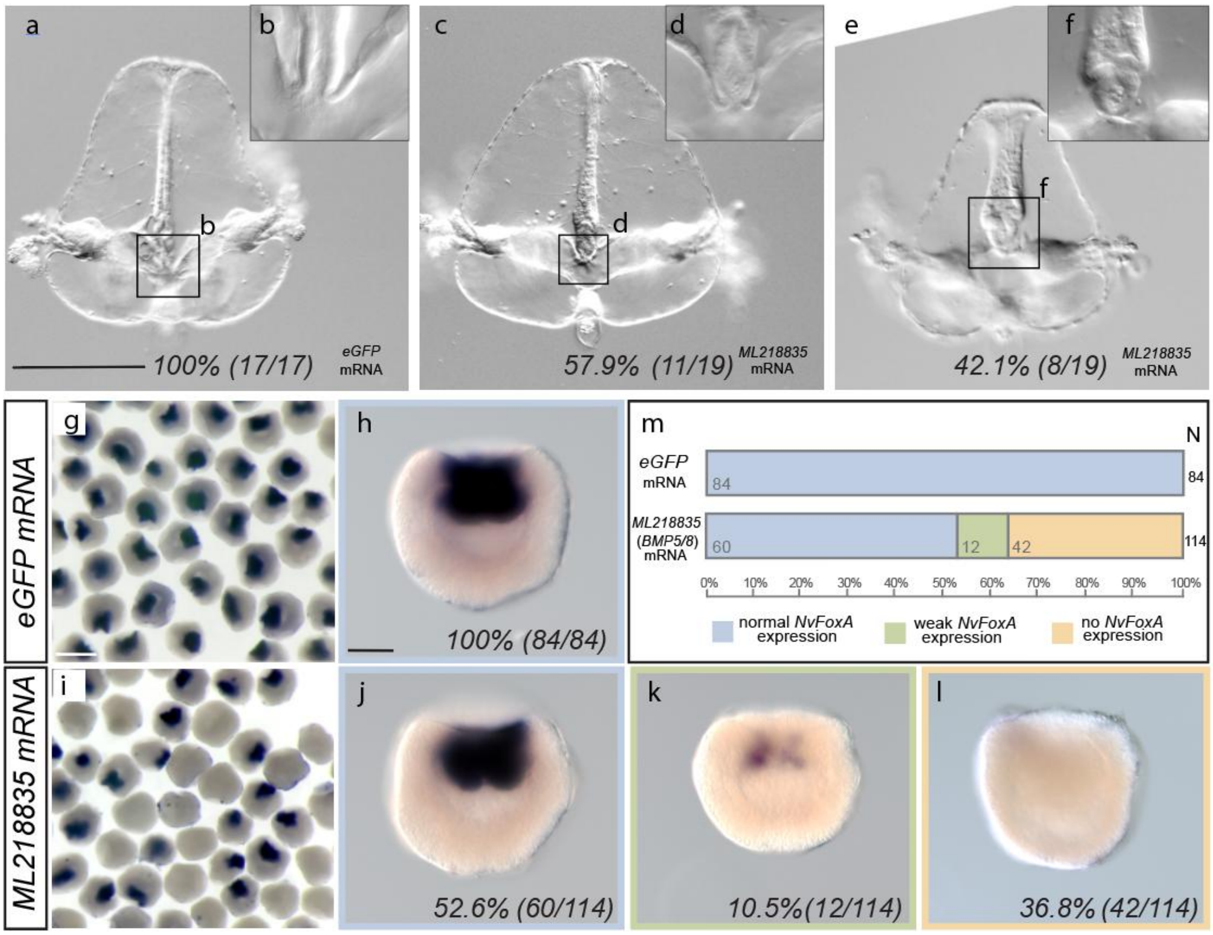

Figure S2: Overexpression of the *M. leidyi* TGFβ ligand *TGFβ_ML218835*, which is expressed aborally during gastrulation, does not induce ectopic oral structure formation in either *M. leidyi* or *N. vectensis*. **a–f**, Microinjection of *M. leidyi TGFβ_ML218835 mRNA* into *M. leidyi* zygotes disrupts pharynx–infundibulum fusion in cydippid larvae **(e, f)**. Control embryos were injected with *eGFP mRNA* **(a)**. **g–m**, Ectopic expression of *M. leidyi TGFβ_ML218835* in a single *N. vectensis* blastomere at the 8-cell stage suppresses *NvFoxA* expression **(i, k–m)**. Control embryos were injected with *eGFP mRNA* **(g, h)**. **(m)** Stacked bars show pooled proportions. Scale bar: **a, c, e**, 250 µm; **g, i**, 200 µm; **h, j-l**, 50 µm. Experiments shown in panels a–f and g–m were independently repeated three times (n=3). The number of samples and the proportion of each phenotype are shown in the panels.
