# Supplementary material for: A blastoporal organizer in a ctenophore
Source: Nature. 2026 Jun 17;655(8124):963–70. doi: 10.1038/s41586-026-10643-z (PMC13391361; doi:10.1038/s41586-026-10643-z)
Supplement: Supplementary file 2 — Reporting Summary [file 41586_2026_10643_MOESM2_ESM.pdf]

nature portfolio

Corresponding author(s): Prof. Andreas Hejnol

Last updated by author(s): 01.04.2026

Reporting Summary

Nature Portfolio wishes to improve the reproducibility of the work that we publish. This form provides structure for consistency and transparency in reporting. For further information on Nature Portfolio policies, see our Editorial Policies and the Editorial Policy Checklist.

Statistics

For all statistical analyses, confirm that the following items are present in the figure legend, table legend, main text, or Methods section.

n/a	Confirmed	
☐	☒	The exact sample size (*n*) for each experimental group/condition, given as a discrete number and unit of measurement
☐	☒	A statement on whether measurements were taken from distinct samples or whether the same sample was measured repeatedly
☐	☒	The statistical test(s) used AND whether they are one- or two-sided<br>*Only common tests should be described solely by name; describe more complex techniques in the Methods section.*
☐	☒	A description of all covariates tested
☐	☒	A description of any assumptions or corrections, such as tests of normality and adjustment for multiple comparisons
☐	☒	A full description of the statistical parameters including central tendency (e.g. means) or other basic estimates (e.g. regression coefficient) AND variation (e.g. standard deviation) or associated estimates of uncertainty (e.g. confidence intervals)
☐	☒	For null hypothesis testing, the test statistic (e.g. *F*, *t*, *r*) with confidence intervals, effect sizes, degrees of freedom and *P* value noted<br>*Give P values as exact values whenever suitable.*
☒	☐	For Bayesian analysis, information on the choice of priors and Markov chain Monte Carlo settings
☒	☐	For hierarchical and complex designs, identification of the appropriate level for tests and full reporting of outcomes
☐	☒	Estimates of effect sizes (e.g. Cohen's *d*, Pearson's *r*), indicating how they were calculated

Our web collection on statistics for biologists contains articles on many of the points above.

Software and code

Policy information about availability of computer code

Data collection	No custom code was used in data collection.
Data analysis	Image processing and analysis were performed using Fiji v.2.14.0. Statistical analyses were performed in R v.4.6.0 using the readxl v.1.4.5, dplyr v.1.2.1, lme4 v.2.0-1, broom.mixed v.0.2.9.6 and emmeans v.2.0.1 packages. Microsoft Excel (Microsoft 365) was used for data handling where appropriate. Figures were assembled using Adobe Illustrator 2026 (v30.3) and Adobe Photoshop 2026 (v27.0). All custom R scripts used for statistical analysis are available in a CodeOcean capsule at https://doi.org/10.24433/CO.6037194.v1 and are provided under an open-source license. No restrictions apply to access or reuse.

For manuscripts utilizing custom algorithms or software that are central to the research but not yet described in published literature, software must be made available to editors and reviewers. We strongly encourage code deposition in a community repository (e.g. GitHub). See the Nature Portfolio guidelines for submitting code & software for further information.

Data

Policy information about availability of data

All manuscripts must include a data availability statement. This statement should provide the following information, where applicable:

- Accession codes, unique identifiers, or web links for publicly available datasets
- A description of any restrictions on data availability
- For clinical datasets or third party data, please ensure that the statement adheres to our policy

All data supporting the findings of this study are available within the paper, its Extended Data and Supplementary Information, as well as in external repositories as detailed below. The gene sequences used for the production of in situ hybridization probes and mRNA synthesis are available in the GenBank database (www.ncbi.nlm.nih.gov/genbank/) under the following accession numbers: AY457634, AF540387, KC137590, AY725201, AY651960, BAH58087, EF427936, DQ988137.1, JF912807, JF912808, JN380186, JN380181.1, JN380180, HM448813, HM448814, HM448815 and HM448816. Data on temporal gene expression during M. leidyi development can be accessed through the M. leidyi Genome Project Portal (https://research.nhgri.nih.gov/mnemiopsis/). Raw replicate-level counts used for statistical analysis are available in CodeOcean at https://doi.org/10.24433/CO.6037194.v1 No restrictions apply to access or reuse.

Research involving human participants, their data, or biological material

Policy information about studies with human participants or human data. See also policy information about sex, gender (identity/presentation), and sexual orientation and race, ethnicity and racism.

Reporting on sex and gender	Not applicable; this study did not involve human participants, human data, or human biological materials.
Reporting on race, ethnicity, or other socially relevant groupings	Not applicable; this study did not involve human participants, human data, or human biological materials.
Population characteristics	Not applicable; this study did not involve human participants, human data, or human biological materials.
Recruitment	Not applicable; this study did not involve human participants, human data, or human biological materials.
Ethics oversight	Not applicable; this study did not involve human participants, human data, or human biological materials.

Note that full information on the approval of the study protocol must also be provided in the manuscript.

Field-specific reporting

Please select the one below that is the best fit for your research. If you are not sure, read the appropriate sections before making your selection.

☒ Life sciences ☐ Behavioural & social sciences ☐ Ecological, evolutionary & environmental sciences

For a reference copy of the document with all sections, see nature.com/documents/nr-reporting-summary-flat.pdf

Life sciences study design

All studies must disclose on these points even when the disclosure is negative.

Sample size	No statistical method was used to predetermine sample size. Sample sizes were chosen based on embryo availability and common practice for these assays.
Data exclusions	Exclusion criteria were pre-established. Entire experiments (datasets), not individual data points, were excluded if viability in the untreated or DMSO control group at 2 dpf was <90%. No other exclusions were applied.
Replication	All experiments were performed in at least three independent biological replicates (independent cohorts of embryos from separate spawn/ collection). All attempts at replication that met the pre-established viability criterion were successful.
Randomization	Formal randomization was not applicable in this experimental design, as embryos were stage-matched, derived from synchronous spawns, and exhibited minimal variability prior to manipulation. Embryos at the appropriate developmental stage were selected and allocated to experimental groups during handling and manipulation, without reference to embryo morphology or any prior phenotypic information. Allocation was performed across embryos from the same spawning cohort.
Blinding	Blinding during data collection or analysis was not applicable due to the visually distinct traits of the studied phenotype.

Reporting for specific materials, systems and methods

We require information from authors about some types of materials, experimental systems and methods used in many studies. Here, indicate whether each material, system or method listed is relevant to your study. If you are not sure if a list item applies to your research, read the appropriate section before selecting a response.

Materials & experimental systems

n/a	Involved in the study
☐	☒ Antibodies
☒	☐ Eukaryotic cell lines
☒	☐ Palaeontology and archaeology
☐	☒ Animals and other organisms
☒	☐ Clinical data
☒	☐ Dual use research of concern
☒	☐ Plants

Methods

n/a	Involved in the study
☒	☐ ChIP-seq
☒	☐ Flow cytometry
☒	☐ MRI-based neuroimaging

Antibodies

Antibodies used: 1:2000 anti-digoxigenin-AP antibody (Roche, 11093274910)

Validation: Other papers that have also used this antibody and found it effective: PMID21931657, PMID27489613, PMID38374160

Animals and other research organisms

Policy information about studies involving animals; ARRIVE guidelines recommended for reporting animal research, and Sex and Gender in Research

Laboratory animals: Wild-type sea anemone Nematostella vectensis were cultured at the Department of Neuroscience and Developmental Biology, University of Vienna. Adult animals of unknown age were used to produce eggs and sperm. Wild-type ctenophore Mnemiopsis leidyi were cultured at the University of Jena. Sex determination is not applicable to M. leidyi as it is a self-fertilizing hermaphrodite. Embryos were obtained from cydippid stage M. leidyi of 1–6 months of age.

Wild animals: No wild animals were collected for this study.

Reporting on sex: Sex was not determined or used as an experimental factor. Analyses were performed on embryos/larvae. M. leidyi embryos were obtained from daily spawnings in laboratory culture; M. leidyi is a self-fertilizing hermaphrodite. N. vectensis embryos were obtained by induced spawning and fertilization. Embryos were pooled and scored without sex-specific stratification.

Field-collected samples: This study did not involve experiments on field-collected samples. All embryos/larvae were obtained from laboratory-maintained cultures (culture/spawning conditions described in Methods).

Ethics oversight: No vertebrate animals or cephalopods were used. The study used non-cephalopod marine invertebrates (Mnemiopsis leidyi and Nematostella vectensis); therefore formal authorization under EU Directive 2010/63/EU (which covers live non-human vertebrates and live cephalopods) was not applicable.

Note that full information on the approval of the study protocol must also be provided in the manuscript.

Plants

Seed stocks: Not applicable; this study did not involve plants or plant-derived materials.

Novel plant genotypes: Not applicable; this study did not involve plants or plant genotypes.

Authentication: Not applicable; this study did not involve plants.
